# Fine Mapping Links the *FTa1* Flowering Time Regulator to the Dominant *Spring1* Locus in Medicago

**DOI:** 10.1371/journal.pone.0053467

**Published:** 2013-01-07

**Authors:** Chin Chin Yeoh, Martin Balcerowicz, Lulu Zhang, Mauren Jaudal, Lysiane Brocard, Pascal Ratet, Joanna Putterill

**Affiliations:** 1 School of Biological Sciences, University of Auckland, Auckland, New Zealand; 2 Institut des Sciences du Végétal, CNRS, Gif sur Yvette, France; Instituto de Biología Molecular y Celular de Plantas, Spain

## Abstract

To extend our understanding of flowering time control in eudicots, we screened for mutants in the model legume *Medicago truncatula* (Medicago). We identified an early flowering mutant, *spring1*, in a T-DNA mutant screen, but *spring1* was not tagged and was deemed a somaclonal mutant. We backcrossed the mutant to wild type R108. The F1 plants and the majority of F2 plants were early flowering like *spring1*, strongly indicating that *spring1* conferred monogenic, dominant early flowering. We hypothesized that the *spring1* phenotype resulted from over expression of an activator of flowering. Previously, a major QTL for flowering time in different Medicago accessions was located to an interval on chromosome 7 with six candidate flowering- time activators, including a *CONSTANS* gene, *MtCO*, and three *FLOWERING LOCUS T (FT)* genes. Hence we embarked upon linkage mapping using 29 markers from the *MtCO/FT* region on chromosome 7 on two populations developed by crossing *spring1* with Jester. *Spring1* mapped to an interval of ∼0.5 Mb on chromosome 7 that excluded *MtCO,* but contained 78 genes, including the three *FT* genes. Of these *FT* genes, only *FTa1* was up-regulated in *spring1* plants. We then investigated global gene expression in *spring1* and R108 by microarray analysis. Overall, they had highly similar gene expression and apart from *FTa1,* no genes in the mapping interval were differentially expressed. Two MADS transcription factor genes, *FRUITFULLb* (*FULb)* and *SUPPRESSOR OF OVER EXPRESSION OF CONSTANS1a* (*SOC1a),* that were up-regulated in *spring1*, were also up-regulated in transgenic Medicago over-expressing *FTa1*. This suggested that their differential expression in *spring1* resulted from the increased abundance of *FTa1*. A 6255 bp genomic *FTa1* fragment, including the complete 5′ region, was sequenced, but no changes were observed indicating that the *spring1* mutation is not a DNA sequence difference in the *FTa1* promoter or introns.

## Introduction

Flowering is a critical step in the life cycle of plants as it heralds the onset of sexual reproduction and the formation of seeds and fruits. The timing of flowering is controlled by environmental cues such as photoperiod and temperature as well as internal signals including developmental age [1]. In eudicots, the genetic regulation of the timing of flowering is best understood in Arabidopsis. In monocots, great progress has been made in rice and the temperate cereals, barley and wheat [2].

In Arabidopsis, a flowering time gene network is involved in perception and response to the signals which are integrated by a set of floral integrator genes [2]. These integrators include *FLOWERING LOCUS T* (*FT*) which encodes a major florigen, the long sought-after universal mobile flowering hormone that in combination with a b-ZIP transcription factor FD, activates pathways leading to the development of flowers [3], [4]. *FT* genes are widespread in plants and many activate flowering, indicating that this function is highly conserved in monocots and eudicots [3]. However, some *FT* genes have other functions, including more general roles in growth [5].

*CONSTANS* (*CO*) plays a key role in promoting flowering in Arabidopsis in long daylength conditions by up-regulating *FT* and over expression of *CO* accelerates flowering [6]–[7]. Rice CO also regulates flowering time, but in this plant it has a more complex dual function depending on the daylength [8]. Other genes, not found in Arabidopsis, also strongly influence flowering in the cereals [9], [10]. On the other hand, a key repressor of Arabidopsis flowering *FLOWERING LOCUS C* (*FLC*) that targets *FT* and other floral integrators appears to be missing from non-Brassicaceous plants [2].

The eudicot legume (Fabaceae) family are the third largest group of plants with commercially significant crop and forage plants such as soybean and alfalfa, and in developing countries providing major staples such as cowpeas, chickpeas and peanuts [11]. It is thus important to understand legume flowering control mechanisms as it is one of the determinants of their performance as a crop in particular geographic locations and climate. Study of flowering in legumes also promises to reveal novel mechanisms of flowering control, because genes such as *FLC,* that are key to Arabidopsis flowering time control, are not found in legume genomes [12], [13], but a similar role might be carried out by another gene. Ultimately, this work should yield new tools for manipulating and customising flowering time using modern plant breeding strategies.

Medicago has a number of attractive features for genetic analysis of flowering time control. There is natural variation in flowering time amongst different Medicago accessions being investigated by Quantitative Trait Locus (QTL) analysis [14]–[16]. For example, a major QTL for flowering time in Medicago has been mapped to an interval on chromosome 7 that contains several candidate flowering regulators, including a *CO-like* gene, *MtCO*, three *FT* genes and an *FD-like* gene [15], [16]. Extensive Medicago mutant resources are available such as *Tnt1* transposon-tagged mutant populations, tilling and fast neutron lines which may be used for forward genetics via screening for flowering time mutants [17]. These mutant populations also provide a powerful opportunity to use reverse genetics to analyse the function of candidate genes mined from the genome sequence and have recently resulted in the identification of a Medicago *FT* gene, *FTa1,* as a regulator of flowering time [18]. In combination with synteny and gene mining in the Medicago genome sequence, work in the related temperate legume, garden pea (*Pisum sativum*) has focused successfully on mutants, many known from classical genetic studies [19]. This work has led to the molecular identification of several flowering time regulators [20], [21], including *GIGAS*, which encodes a pea florigen *FTa1* [22].

To extend our understanding of flowering time control in eudicots, we aim to carry out forward screens for Medicago flowering time mutants as a prelude to functional gene characterization. Here we report that we screened a Medicago T-DNA mutant population [23] and identified an early flowering mutant that we named *spring1*. *Spring1* is not T-DNA tagged and thus can be classed as a somaclonal mutant. However, we show that *spring1* behaves as a single dominant Mendelian gene that confers early flowering. We mapped *spring1* to an interval of ∼0.5 Mb on chromosome 7. This interval contains 78 predicted genes, including the three *FT* genes, but not *MtCO* or other known candidate flowering time genes. Only one of the *FT* genes, the floral regulator *FTa1*, was up-regulated in *spring1* mutants, and no changes, either up or down, were observed to expression of the other genes in the interval by microarray analysis. These results strongly indicate that it is the increased abundance of *FTa1* that is causing the *spring1* early flowering phenotype.

## Materials and Methods

### Plant Material and Growth Conditions

Jester [24] and R108_C3 (R108) [25] are two genotypes belonging to two subspecies of *Medicago truncatula* Gaertn (barrel medic), *ssp. truncatula* and *ssp. tricycla* respectively. Jester is an aphid-resistant line closely related to Jemalong/A17. The *spring1* mutant was identified during a glasshouse mutant screen (Institut des Sciences du Végétal, CNRS, Gif sur Yvette, France) of a R108 T-DNA tagged population [23]. The *35S::FTa1* transgenic Medicago lines in the R108 genotype were previously reported [18].

Plants for all subsequent flowering time experiments and gene expression experiments (with the exception of the diurnal timecourse where plants were grown in sterile conditions in plant growth cabinets as described previously [18]) were grown under long-day conditions (LD, 16 h light/8 h dark) in a growth room with ∼200 µM m^−2^ s^−1^ cool white fluorescent light at ∼22°C. For germination, seeds were scarified by gently rubbing them between two pieces of sand paper (grade P160) until small signs of abrasion appeared. Scarified seeds were incubated at 4°C on 0.8% water agar in the dark for 3 days to overcome embryonic dormancy, and then left at 22°C in the dark for another 4 to 5 hours to complete germination. Germinated seedlings were grown in a soil mix consisting of 9 parts of Black Magic® seed raising mix (Yates, Orica New Zealand Ltd.), 3 parts of coarse graded vermiculite (Pacific Growers Supplies Ltd.) and 1 part of No. 2 Propagating Sand (Daltons Ltd.). They were watered with tap water and a complete liquid nutrient media [26].

### Plant Crosses and Scoring Flowering Time

Plant crosses were carried out to investigate the genetics and inheritance of *spring1* on the one hand and to develop populations for mapping *spring1* by linkage analysis with DNA markers on the other. Multiple crosses between each genotype were done, in both directions, using *spring1* as a male or as a female, under a binocular microscope by emasculating the female plants, dusting pollen from the male plant over the stigma and then wrapping the pollinated flower in plastic film for three days [27]. Four types of crosses were made; Backcrosses between *spring1* and R108, Control crosses between the two wild type genotypes, R108 and Jester, Mapping crosses between *spring1* and Jester and Test crosses between the F1 plants from *spring1* x Jester and Jester. These crosses are described in more detail below and flowering time results are presented in Table 1.

**Table 1:** Flowering time of plant populations derived from crosses with the *spring1* mutant.

Genotype	Average Number of Days after Germination to Flowering	Day Range	Average Nodes on PrimaryAxis at Flowering	Node Range	Flowering Time		Total No. Plants
					No. Early Plants	No. Late Plants	
**Parental lines**							
R108	49.8+/− SE 0.13	49–50	12.9+/− SE 0.31	12–16	0	12	12
Jester	37.5+/− SE 0.92	35–41	12.2+/− SE 0.31	11–13	0	6	6
*spring1*	25.3+/− SE 0.25	25–28	6.6+/− SE 0.26	6–9	12	0	12
**Backcross “** ***spring1*** ** x R108”**							
F1 (♂*spring1* x ♀R108)	29+/− SE 0	29	7.4+/− SE 0.21	6–8	27	0	27
Early F2 (♂*spring1* x ♀R108)	24.7+/− SE 0.21	24–31	7.3+/− SE 0.17	6–10	62	0	78
Late F2 (♂*spring1* x ♀R108)	54.1+/− SE 0.72	49–59	nd	nd	0	16	
**Mapping Cross “** ***spring1*** ** x Jester”**							
F1 (♂*spring1* x ♀Jester)	32.2+/− SE 0.64	31–39	7.7+/− SE 0.37	6–9	32	0	32
Early F2 (♂*spring1* x ♀Jester)	25.3+/− SE 0.27	16–57	6.4+/− SE 0.04	4–9	421	0	747
Late F2 (♂*spring1* x ♀Jester)^a^	–	>39– >87	–	11– >25	0	57	
Unclassified F2 (♂*spring1* x ♀Jester)^b^	–	28– >73	–	8– >10	94		
Dead F2 (♂*spring1* x ♀Jester)	–	–	–	–	175		
**Control Cross “R108 x Jester”**							
F1 (♂R108 x ♀Jester)	60.2+/− SE 4.83	44–78	13.6+/− SE 0.60	11–16	0	12	12
F2 (♂R108 x ♀Jester)^c^	–	52– >65	–	11–>19	0	12	18
Unclassified F2 (♂R108 x ♀Jester)	53.7+/− SE 2.03	50–57	8.3+/− SE 0.67	7–9	3		
Dead F2 (♂*spring1* x ♀R108)	–	–	–	–	3		
**Mapping “Test Cross”**							
Early Test Cross	22.4+/− SE 0.34	18–35	7.2+/− SE 0.08	5–9	83	0	275
Late Test Cross^d^	–	38– >69	–	11– >21	0	95	
Unclassified Test Cross^e^	–	30– >69	–	8– ≥11	30		
Dead Test Cross	–	–	–	–	67		

Flowering time was scored and plants were classified for flowering time. Plants were classified as early flowering if they had ≤7 nodes at flowering or had flowered rapidly (≤28 days after germination), similar to *spring1*. They were classified as late flowering if they had ≥11 nodes at flowering or had not yet flowered but had ≥11 nodes, similar to Jester. They were scored as unclassified due to not falling into our two classes, or being difficult to score due to their tiny size or altered aerial architecture.

a, b F2 plants were grown in six groups and scoring was terminated after 39, 46, 73, 77, 83 or 87 days.

a A total of 27 plants had not yet flowered when scoring was terminated. None of the plants classified as late had flowered by 39 days. Four plants had not flowered by 87 days and had up to 25 nodes on the primary axis.

b In total 10 plants had not flowered by the time scoring was terminated.

c F2 plants were grown up and scoring was terminated after 65 days. Two plants had not yet flowered and had up to 19 nodes on the primary axis.

d/e Scoring was terminated at 69 days.

d A total of 33 plants had not yet flowered and had up to 21 nodes on the primary axis.

e Two plants had not yet flowered, one had 10 nodes and one was unscoreable due to its small size and architecture.“Test Cross” is (♂(♂*spring1* x ♀Jester) x ♀Jester). Plants classified as Dead, usually died as very young seedlings. nd is not done.

#### The backcross

We backcrossed *spring1* with wild type R108 plants to investigate the genetics and inheritance of *spring1*. The seed from the crosses were collected and we planted out F1 plants, scored their flowering time which was early, similar to *spring1*, and then allowed these plants to self-fertilise to produce the F2 generation. The F2 generation was then sown out and scored for flowering time. No differences in phenotype were observed when *spring1* was used as a male or a female plant in the backcross to wild type. Data from the cross of *spring1* as a male and R108 as a female is presented. Flowering time measurements were carried out by recording the days after germination to the first floral bud, and/or by counting the node number on the primary axis of each plant at flowering. Plants segregating for flowering time were classified as early (*spring1*-like) or late flowering (R108-like) in order to determine the segregation ratio.

#### The control cross

In order to examine the effect on flowering time and other plant phenotypes of crossing the two genotypes, R108 and Jester, we crossed R108 with Jester. The seed from the crosses were collected and we planted out the F1 plants, scored their flowering time, which was late, and then allowed these plants to self-fertilise to produce the F2 generation. The F2 seed was sown out and scored for flowering time. No differences in phenotype were observed when R108 was used as a male or a female plant in the cross to Jester. However, a feature of the crosses of R108 to Jester, was that the parental and progeny plants grew at different rates. Hence, the number of nodes on the primary axis at flowering, rather than days after germination to flowering, was selected as the most accurate way of scoring the flowering time in the control cross. All of the F1 progeny were classified as late flowering as they had ≥11 nodes at flowering, similar to Jester and R108. The F2 progeny were classified as late flowering as they had ≥11 nodes at flowering, similar to Jester and R108, or unclassified due to being difficult to score due to their tiny size or altered aerial architecture.

#### The mapping cross

To develop a population for mapping *spring1* by linkage analysis with DNA markers, *spring1* was crossed to Jester. We used *spring1* either as the male or as the female plant. The seed from the crosses was collected and we planted out the F1 plants, scored their flowering time, which was early, similar to *spring1*, and then allowed these plants to self-fertilise to produce the F2 generation. The F2 seed from five different F1 plants was then sown out and scored for flowering time. No differences in phenotype were observed when *spring1* was used as a male or a female plant in the backcross to Jester. Due to lack of space, we grew the F2 plants in batches with *spring1*, Jester or R108, which confirmed that the control plants flowered at a similar time in each experiment. Data from the cross of *spring1* as a male and Jester as a female is presented. F2 plants were classified as early flowering if they had ≤7 nodes at flowering or had flowered rapidly (≤28 days after germination), similar to *spring1*. The F2 progeny were classified as late flowering if they had ≥11 nodes at flowering, similar to Jester. A third group F2 plants were categorised as unclassified, due to not falling into our two classes, or being difficult to score due to their tiny size or altered aerial architecture.

#### The test cross

In order to further analyse the inheritance of *spring1* and to generate more plants for linkage analysis, we carried out a Test cross between F1 plants from “*spring1* x Jester” as the male and Jester as female. Eighty crosses were carried out. Barrels from the crosses were collected and progeny plants were grown up and classified as early flowering if they had ≤7 nodes at flowering or had flowered rapidly (≤28 days after germination) similar to *spring1*, or late flowering if they had ≥11 nodes at flowering, similar to Jester. A third group of progeny plants were categorised as unclassified, due to not falling into our two classes, or being difficult to score due to their tiny size or altered aerial architecture.

### *Spring1* Linkage Mapping with DNA Markers

We tested if *spring1* co-segregated with DNA markers from BACs in the region of chromosome 7 that had been previously shown to contain a major QTL for flowering and carried six candidate activators of flowering [15]. The candidate activators were *MtCO,* Medtr7g083540; *FTa1,* Medtr7g084970; *FTa2,* Medtr7g085020 and Medtr7g085030; *FTc,* Medtr7g085040; *FD*, Medtr7g088090 and *PHYTOCHROME KINASE SUBSTRATE I* (*PKS),* Medtr7g088200. We carried out the linkage analysis on the F2 plants from the Mapping cross of *spring1* x Jester and on the progeny of the Test Cross. For example, a marker that was closely linked to *spring1* would be expected to co-segregate close to 100% with flowering time in the following way: The *spring1* version of the marker would be present in the early-flowering segregants (homozygous or heterozygous in the F2 plants and heterozygous in the Test cross) and the Jester version homozygous in the late-flowering plants. Recombination events between a closely-linked marker and *spring1* were detected as follows: Recombinants in the late flowering F2 class from the “*spring1* x Jester” cross were identified by plants that were heterozygous for the Jester allele. The recombinant plants amongst the early flowering F2 plants, that could be distinguished, were homozygous for the Jester allele. In the Test cross, recombinants in the late flowering class were heterozygous for the Jester allele and in the early flowering class they were homozygous for the Jester allele.

The primer sequence of existing markers were obtained from the Integrated Genetic Map of *Medicago truncatula* http://www.medicago.org/genome/map.php or from Pierre *et al* [15]. New insertion/deletion (indel) or Simple Sequence Repeat (SSR) markers were also developed. Primers that flanked introns or that encompassed SSRs were tested for the ability to detect polymorphisms. When DNA sequence annotation was not provided for a BAC from Genbank, a Chromosome Visualisation Tool (CViT) BLAST search http://medicagohapmap.org/with the BAC sequence against the Medicago pseudomolecule Mt3.5 genome assembly was carried out. This provided GeneCall Identities of all the genes in the BAC “Mt3.5 BAC Genecall Table”. By searching with a GeneCall ID against the TIGR/JCVI GBrowse “Medicago GBrowse- IMGAG Annotation v3.5” http://gbrowse.jcvi.org/cgi-bin/gbrowse/medicago/#search, a detailed gene model provided annotation of predicted coding sequences, 5′ and 3′ untranslated regions. Additional SSR markers in non-annotated BACs were identified using a Geneious http://www.geneious.com/plug in “Phobos” http://www.ruhr-uni-bochum.de/spezzoo/cm/cm_phobos.htm.

### DNA Extraction and PCR Amplification

Genomic DNA from plants was extracted with the Extract-N-Amp™ Plant PCR Kit (Sigma-Aldrich New Zealand Ltd.) on ∼0.5 cm diameter disks of young leaf tissue. The guidelines of the manufacturer were followed but the volume of Extraction Solution and Dilution Solution used per sample was halved. For PCR, 1 µL of the extracted genomic DNA was used in a 10 µL reaction containing 1× Phire® Reaction Buffer, 0.2 mM dNTPs, 0.2 µM of each primer and 0.2 µL of Phire Hot Start II DNA Polymerase. Genomic DNA was omitted for control reactions. The primer sequences are given in Table 2. The PCR reaction was carried out on an Applied Biosystems 96-well GeneAmp® PCR System 9700 machine. The reaction program consisted of an initial denaturation step at 98°C for 30s, followed by 35 cycles of denaturation at 98°C for 10s, annealing at 55°C for 10s and elongation at 72°C for 15 s/kb depending on expected amplicon size, and a final elongation step at 72°C for 1 min. The reaction was then cooled down to 15°C. PCR products were separated on a 3% agarose gel or on a 12.5% polyacrylamide gel.

**Table 2:** DNA markers used for fine mapping *spring1* on chromosome 7.

Marker	BAC	Mt3.5 Genome assembly	UMN (cM)	Type	Forward Primer	Reverse Primer	cBand Size (bp)	dLate F2	dEarly F2	dLate Test Cross	dEarly Test Cross
ah2_9|7d	CT967302	chr7:17913549..17913763	42.4	SSR	AAGGGCCACAAAGAGAAGGT	GGTGGAATCAAAGCACCAAT	215	7/57	NT	NT	NT
ah2_172c4a	AC146784	chr7:19580059..19580346	47.0	SSR	GCCGAATATGCGAGCTTTT	TGGCTCACCTTCCACTTCA	288	6/57	NT	NT	NT
a003B12	AC159114	chr7:21245026..21245168	47.7	SSR	GCTTTTGAGGTCAAAGTACAA	CAGGGAGTAGCTTGTAAATCA	143	4/57	NT	NT	NT
ah4_42i13a	AC161750	chr7:21396080..21396350	47.7	SSR	TGTATGCCAAGCATCGGTTA	TGCCAAGAGGAAACTTGGTT	271	4/57	NT	NT	NT
aH2_142N19_fr1	AC147179	chr7:22856921..22857163	50.4	SSR	CCAATTTTGACACCCTACAT	GAGATTAAATCCACCCCTTC	243	1/57	NT	NT	NT
Medtr7g080490	AC147179	chr7:22879847..22880323	–	indel	GCCAGCTCCAGCTGACATTGTGG	CCCTTGAGGTCAGTCTCAACCGT	477	1/57	3/419	1/95	3/83
ah2_14c17b	AC142095	chr7:23179073..23179192	50.4	SSR	AGCTGCTCTCAGTGCCATTT	TGCGTGTGACAAGTTCCTCT	120	0/57	NT	1/95	2/83
a003A09	AC133780	chr7:24672851..24672966	50.4	SSR	TTGTGGTGACTAGTGATTGG	ATGTGAAGTAAATCCCTTGC	116	0/57	3/419	0/95	0/83
MtCO	AC133780	chr7:24688717..24688738	–	indel	GAATAGAGGGGATGCCATGTT	CAAATCGCCCTCTCACTCTC	381	NT	3/419	NT	NT
TF_B3	AC149131	chr7:24783230..24783575	–	indel	TCCGACTGTTATTGTTAGGCCA	TGCAGAAAGCAGCAGAGGCT	346	NT	2/419	NT	NT
eIF-4gamma	AC149131	chr7:24840562..24840940	–	indel	TCTGGCTATTGTGAGATGGGTCT	ACGGTTGTATGTTTTGCTTACTGC	379	NT	1/419	NT	NT
Medtr7g084090.1_SUVH4	AC155894	chr7:24973864..24974348	–	indel	TCCAGGACGCTATCAAGCGCA	CGAGTGGTGCTGTTTGCCGC	485	NT	1/419	NT	NT
**Medtr7g084170.1**	AC186135	chr7:25016694..25017139	–	indel	AGGAAATTCTACCAAAGATTCTGAG	TCATCCAAGTTTGACCGTTTTCT	446	NT	1/419	NT	NT
Medtr7g084560.1	AC157890	chr7:25212756..25213142	–	indel	AGCATTGACAGGATTTCGTGATGC	TGGCCACTACGATCCAGCTCA	387	NT	0/419	NT	NT
FTa1	AC123593	chr7:25433850..25434142	–	indel	CCTTCAAAATATGAAAAAGGGCTA	AAATTTAAAATGTCTTCCTTGCTC	293	0/57	0/419	0/95	0/83
Medtr7g085120	AC123593	chr7:25499863..25500262	–	indel	GCTGGTAGCACTGGTAGCCGTA	GCAGCTTGTTCCGGATCATTTGCAT	400	0/57	NT	0/95	0/83
**Medtr7g085190.1**	AC145753	chr7:25527032..25527377	–	indel	TGAGGCTTATTGTTCAAGTCAAGGA	TGGTAAACCTTATGCTGAGAGGA	346	1/57	0/419	0/95	0/83
Medtr7g085200	AC145753	chr7:25537421..25538020	–	indel	CCCAAGTTTCCCAAAACATAGT	GGTTTTGGTTTCATGAGAGATGGT	600	1/57	NT	0/95	4/83
AC145753_RR1	AC145753	chr7:25593027..25593223	–	SSR	ACGAGGAACGGTGGCAGAAGGA	TGGTTGGTCTTGTAATATTGGC	197	2/57	NT	NT	NT
AC167711_RR8	AC167711	chr7:25677751..25677868	–	SSR	TGCTGAGGCACGTCCCAGTG	ACCCCTGAAACCAGACAAGCCA	118	3/57	NT	NT	NT
AC167711_RR5	AC167711	chr7:25695231..25695594	–	SSR	GTGAAGAGCAAGGGGTCGCGT	TGATACAACAAAGACAAACCACAGGCT	364	3/57	NT	NT	NT
AC148816_RR1	AC148816	chr7:25943053..25943342	–	SSR	TCAGCTTGGGAACGTTGGTCGT	GGAGGCGCATCATCACCCCG	290	4/57	NT	NT	NT
AC148816_RR4	AC148816	chr7:25952355..25952590	–	SSR	GGGTCATGGCCTTGGACCACA	ACCTCCTCCAACAAAAGCCACCT	236	4/57	NT	NT	NT
AC148816_RR5	AC148816	chr7:25957056..25957337	–	SSR	AGACGATGATGAAGGTGAGGATGGA	TCAGCCTTCTTTTCCAAGAGTGCTTC	282	4/57	NT	NT	NT
AC148816_RR12	AC148816	chr7:26039826..26040091	–	SSR	TGGCTGAATAACTGTTGTGCAAGGCT	TCAGTGGACGATCGTTGATACTTGTG	266	4/57	NT	NT	NT
a002E05	AC126009	chr7:26164550..26164669	53.3	SSR	ATGGAAGGTGGAACCTATCT	GGTGTCGACTGATCCTAGC	120	6/57	NT	NT	NT
a005G08	AC141107	chr7:27227337..27227598	53.3	SSR	GGTTTACTGGCCCTCAACAA	CTCCGTATGCCTTTCTTCCA	262	13/57	NT	NT	NT
ah2_81g19a	AC153128	chr7:28185897..28186094	56.1	SSR	GTTCCAAAAACGCACCAAGT	CATGACAGCAGTACATTGCC	198	20/57	NT	NT	NT
bMTIC714	AC126016	chr7:34518760..34518883	57.0	SSR	TAGAAAAGCACAACAAGCTG	TGCTACGTATCAAATCAACAA	124	29/57	NT	NT	NT

DNA markers from the *MtCO* region of chromosome 7 were analysed for linkage to *spring1* in the mapping crosses (see Text). The interval containing *spring1* is flanked by Medtr7g084170.1 and Medtr7g085190.1 shown in bold; each marker is separated from *spring1* by one recombination event.

a DNA marker taken from the University of Minnesota (UMN) Integrated Genetic Map of *Medicago truncatula* http://www.medicago.org/genome/map.php.

b DNA marker from Pierre *et al* (2010).

c Size of PCR fragment predicted from Mt3.5 Genome assembly from http://medicagohapmap.org/.

d Numbers of recombinants detected by the marker in the late or early flowering classes from the F2 of the “*spring1* x Jester” cross, or from the Test cross (see Table 1 and Text). “Test Cross” is (♂(♂*spring1* x ♀Jester) x ♀Jester). NT is not tested. Indel is insertion/deletion; SSR is Simple Sequence Repeat. The column entitled Mt3.5 Genome assembly gives the position of the marker on the pseudomolecule from Mt3.5 Genome assembly from http://medicagohapmap.org/after a Chromosome Visualisation Tool (CViT) BLAST search http://medicagohapmap.org/was carried out.

### Analysis of Gene Expression by qRT-PCR

RNA extraction, cDNA synthesis using an oligo dT primer and qRT-PCR on an Applied Biosystems 7900HT Sequence Detection System was carried out as previously described [18]. Each data point presented is derived from two or three biological replicates harvested in parallel, with each replicate consisting of a pool of tissues from at least three independent plants. All qRT-PCR results were replicated in one or more experiments on independently grown plants. Whole aerial parts of plants with three fully-expanded true leaves were analysed, or leaf and shoot apical samples were harvested separately, as described in the text. The PCR primer sequences used were as previously described for *FTa1*, *FTa2*, *FTc, Tubulin* (*TUB*) and *PROTODERMAL FACTOR 2* (*PDF2*) [18]. The other primer sequences used for qRT-PCR are listed in Table S3. The identity of PCR amplicons were confirmed by DNA sequencing.

### Microarray Analysis of Global Gene Expression

Total RNA was extracted from R108 and *spring1* grown in long daylength conditions from the first trifoliate leaf when seedlings were 12–14 days old and at the three true-leaf stage (one monofoliate leaf and two trifoliate leaves). Three biological replicates were harvested in parallel, each consisting of a pool of leaves from three independent plants. RNA quality was checked using RNA 6000 Nano Chip using the Agilent 2100 Bioanalyzer instrument. cDNA synthesis from each of the biological replicates, labelling and hybridisation to the Affymetrix *Medicago* GeneChip arrays were performed according to the manufacturer’s instructions (Affymetrix, Santa Clara, CA, USA).

Statistical analysis of microarray data was performed using Bioconductor in the R statistical computing environment (http://www.R-project.org). Briefly, normalization was performed using the Robust Multichip Algorithm (RMA) with background correction. Normalized data were then analyzed using the limma package [28] to identify differences in expression levels between the genotypes. Differentially-expressed genes were selected based on statistical significance (false discovery-rate (FDR), corrected *p*-values of ≤0.05 and fold-change magnitude (2-fold or greater up or down).

### DNA Sequence Analysis of the *FTa1* Genomic Region

PCR was used to amplify 6346 bp of the *FTa1* genomic region from *spring1* and R108 from the nearest upstream gene Medtr7g084960 to 511 bp downstream of the translation termination codon. The primers used were: 5′- TGCAAACATAGAAAGGCCATC -3′ and 5′- TGTTTGTGGTTTGCAGCAGT -3′. The resulting PCR fragments were directly sequenced, DNA sequence contigs assembled using Geneious software http://www.geneious.com/ and compared with each other and with the Jemalong/A17 sequence. Alignment of the 5′ region was done using the MultAlin program [29]. The R108 and *spring1* sequences were identical, but differed from A17. The accession number of the R108 sequence is GenBank KC108841.

## Results and Discussion

### *Spring1* Confers Dominant Early Flowering

**Figure 1:**
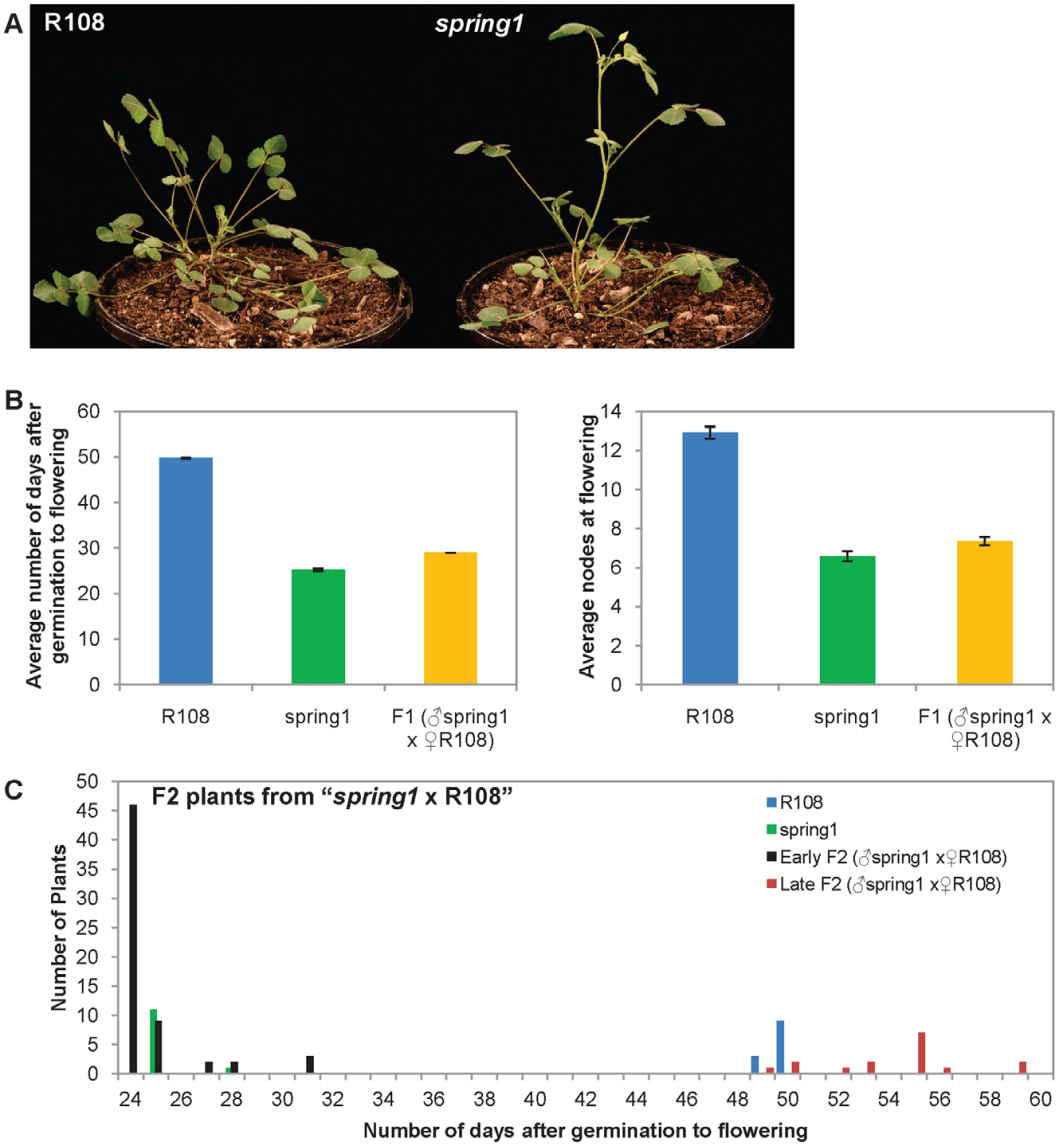
Flowering time of plants from the Backcross “*spring1* x R108”. *Spring1*, an early flowering mutant, was backcrossed with wild type R108 plants and the F1 and F2 progeny were grown in long day conditions and scored for flowering time. a) Photographs of R108 wild type plant and the *spring1* mutant plants. Both plants were photographed 30 days after germination. b) Flowering time of the F1 progeny (n = 27) compared to *spring1* (n = 12) and R108 (n = 12). The F1 plants flowered much more rapidly than R108 and at a similar time to *spring1*. Similar results were obtained when flowering time was scored using either of two methods; the number of days after germination to flowering, or the number of nodes on the primary axis at flowering. c) Distribution of the flowering time of the F2 progeny compared to *spring1* and R108. The F2 population segregated 62 early flowering and 16 late flowering plants, as scored by days after germination to flowering, and by comparison to the parental lines, indicating that *spring1* was a monogenic dominant mutation.

In order to identify genes that regulate flowering in Medicago, we carried out a glasshouse screen of a T-DNA tagged population in the R108 accession [23]. An early flowering mutant was identified that we named *spring1* (Fig. 1a). However, the mutant was not T-DNA tagged as determined by PCR genotyping or Southern blot analysis with T-DNA sequence probes (data not shown). Thus *spring1* can be deemed a somaclonal mutant and is likely to have arisen during plant regeneration [30]. In Medicago, two genes that were tagged with an endogenous MERE1-1 retroelement were identified in somaclonal mutants [31], [32]. MERE1-1 is a low copy copia-type retroelement that is active during regeneration of Medicago in tissue culture [31]. Thus, we carried out transposon display experiments to identify MERE1-1 insertions in *spring1*, but none of the six insertions obtained were linked to the *spring1* early flowering phenotype (data not shown).

In order to investigate the genetics and inheritance of *spring1*, we backcrossed it to wild type R108 plants and grew the plants under long daylength conditions (Fig. 1, Table 1). All the F1 plants flowered significantly earlier than R108, at a similar time to *spring1*, when flowering time was measured both as the number of days after germination to flowering, or as the number of nodes on the primary axis at the time of flowering (Fig. 1b; Table 1). Next, we allowed the F1 plants to self fertilise, planted out the resulting 78 F2 plants and scored the number of days after germination to flowering. The F2 plants segregated into two groups, with the majority flowering early like *spring1* (Fig. 1c; Table 1). The flowering time of the F1 and F2 plants strongly indicate that *spring1* confers dominant early flowering time. The experimental hypothesis of a single dominant gene is further supported by an approximate 3∶1 segregation ratio observed in the F2, as 62 F2 plants flowered early while 16 were late (χ^2^ = 0.84; 0.1<p<0.5).

### Crosses of *spring1* and R108 to Jester

We carried out two crosses to develop populations for mapping *spring1*. These were the Mapping cross and the Test cross, both of which involved crossing *spring1* with Jester. We also carried out a Control cross between R108 and Jester. Jester is closely related to Jemalong/A17 (ssp. *truncatula*), the subject of the Medicago genome sequencing project [13], [24]. R108 is the background genotype of *spring1*, but is a different subspecies from Jester, ssp. *tricycla* [33]. While the cross between the two Medicago subspecies provides abundant polymorphisms for mapping, a disadvantage is that it also affects plant growth. For example, previously, chlorosis in F1 plants was observed in a cross of R108 × A17 [33]. Therefore, the Control cross was also done between wild type R108 and Jester to enable us to test the hypothesis that a seedling’s reduced growth was not due to *spring1* and that flowering time was not affected in progeny of crosses between Jester and R108 genotypes. Developmental difficulties were not reported in the mapping populations previously used to identify Medicago flowering time QTLs, but these were carried between Jemalong and other accessions of the same sub species (*ssp. truncatula*) [16].

**Figure 2:**
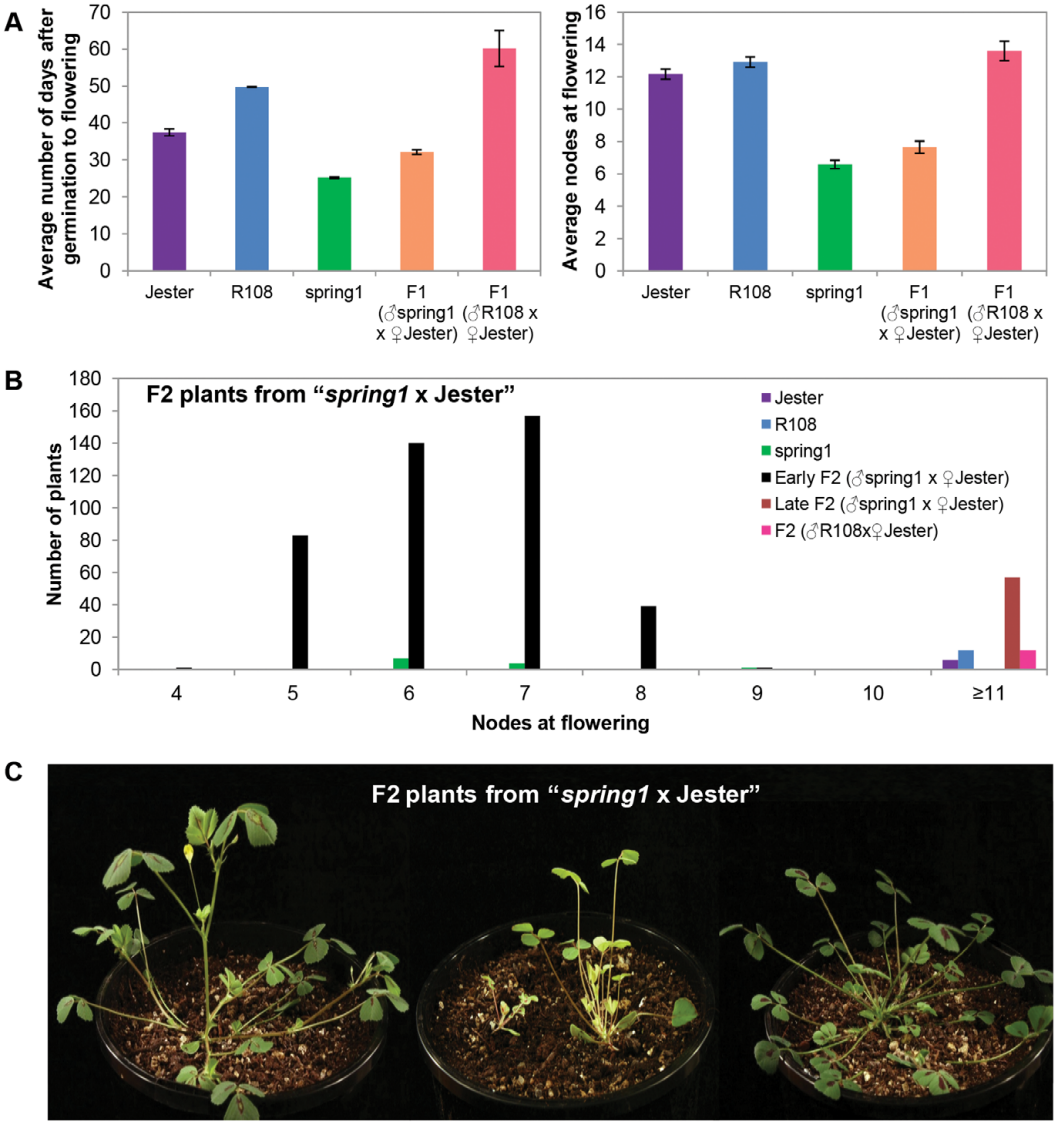
Flowering time of plants from “*spring1* x Jester” and from “R108 x Jester”. *Spring1*, an early flowering mutant in the R108 accession was crossed with Jester plants and the F1 and F2 progeny were grown in long day conditions and scored for flowering time (Table 1). A Control cross “R108 x Jester” was also performed. a) Flowering time of the F1 progeny from the Backcross “*spring1* x Jester” (n = 32) and from the Control cross “R108 x Jester” (n = 12) was compared to *spring1* (n = 12), Jester (n = 6) and R108 (n = 12). Flowering time was scored using two methods; the number of days after germination to flowering, or the number of nodes on the primary axis at flowering. The F1 plants from the Mapping cross flowered much more rapidly than the F1 plants from the Control cross by either measure, indicating that *spring1* confers dominant early flowering in crosses to Jester. b) Distribution of the flowering time of the F2 progeny from the Mapping cross and the Control cross compared to parental lines. Plants that were scored as “unclassified” or died young are not included. The F2 population from “*spring1* x Jester” segregated 421 early flowering and 57 late flowering plants as scored by nodes at flowering. The class with ≥11 nodes includes plants that had up to 25 nodes, but had not flowered by the time scoring was terminated at 87 days. The Control cross produced only late flowering F2 plants, with some having up to 19 nodes, but not having flowered by the time scoring was terminated at 65 days. c) Photographs of F2 plants from the “*spring1* x Jester” Mapping cross; a typical early flowering plant with flowers (left), plants that have not flowered that are either very small, pale and slow growing, or small with an altered morphology (middle), and a typical late flowering plant (right). All plants were photographed at 26 days old.

The F1 plants from the crosses between *spring*1 and Jester and the cross between R108 and Jester grew more slowly and were smaller and paler than the parents. Because of the variation in growth rates between the parents and the F1 progeny, we elected to score the flowering time of progeny from these crosses, primarily based on the number of nodes to flowering. We reasoned that using the alternative method to score flowering time, in days after germination to flowering, could be misleading as slow growth might result in a plant being classified erroneously as late flowering. The “*spring1* x Jester” F1 plants flowered early and at a similar node number as *spring1* plants and much earlier than plants from the control cross “R108 x Jester” which flowered late like Jester (Fig. 2a, Table 1). This confirms that *spring1* confers dominant early flowering in the cross to Jester, as it does to R108.

Next, groups of F2 plants from “*spring1* x Jester” (747 total plants), and a small F2 population of 18 plants from the control cross “R108 x Jester”, were grown up. A total of 175 (23.4%) “*spring1* x Jester” and 3 (16.6%) “R108 x Jester” F2 plants died (Table 1). Such effects on growth and seedling mortality were observed before in crosses between plants in R108 and Jemalong backgrounds [33]. F2 plants were classified as early flowering if they had ≤7 nodes at flowering or had flowered rapidly (≤28 days after germination), similar to *spring1*. They were classified as late flowering if they had ≥11 nodes at flowering, similar to Jester (Fig. 2b, Table 1). We scored 421 and 57 plants as early and late flowering, respectively. A third group of 94 F2 plants remained unclassified (Table 1), due to not falling into our two classes, or being difficult to score due to their tiny size or altered aerial architecture (Fig. 2c). The phenotypic distribution of flowering time was broader amongst the late flowering class in the F2 than seen in the parental Jester plants (Table 1). For example, the late class of F2 plants from “*R108* x Jester” flowered with 11->19 nodes, while Jester flowered with 10–14 nodes. This may stem from interactions between the two genotypes. From a total of 18 F2 plants grown from the control cross “*R108* x Jester”, 12 flowered late and 3 were unclassified (Table 1).

In total, 421 of the 572 surviving plants were classified as early flowering, which is consistent with *spring1* conferring dominant early flowering (ie. expected 3∶1 ratio for early:late flowering). However, of the remaining plants, we could only confidently score 57 as late flowering. This gives a ratio of early-flowering to late-flowering plants highly skewed toward early flowering, with a ∼7∶1 ratio (χ^2^ = 43.6; p<0.001). This observed ratio does not support the experimental hypothesis of a monogenic dominant gene using this F2 progeny. Results of complementary genotyping experiments indicate that there is segregation distortion in the F2 (Table S1) and this was also observed in the Recombinant Inbred Line populations used to map the major QTL for flowering on chromosome 7 [14]–[16].

**Figure 3:**
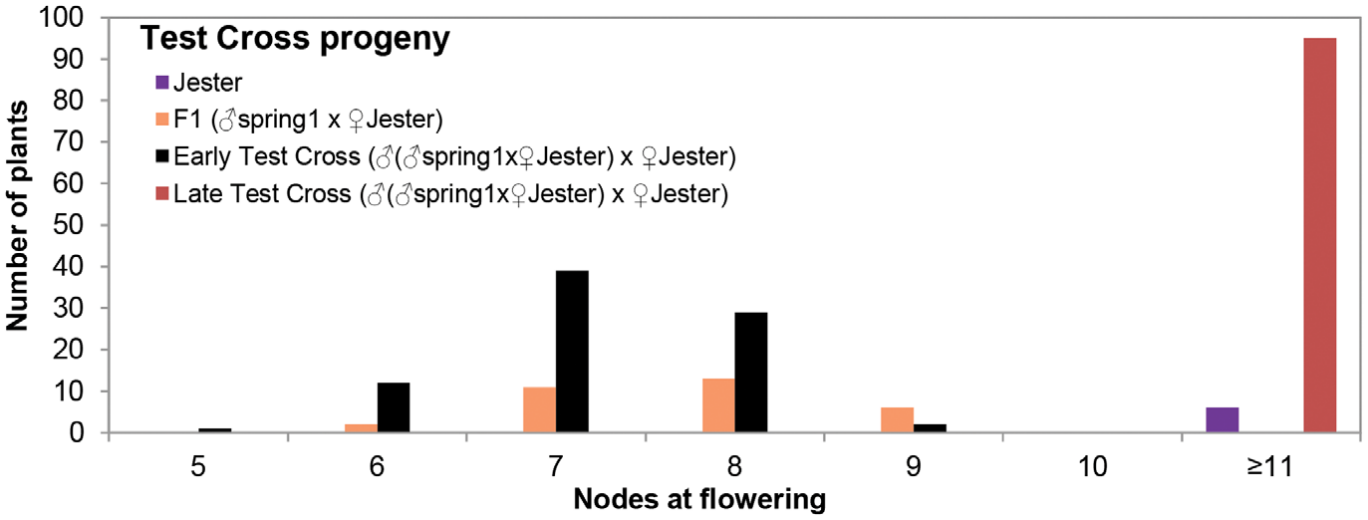
Flowering time of plants from the Test cross. *Spring1*, an early flowering mutant, was crossed with Jester plants and the resulting F1 plants were then crossed with Jester in the Testcross (♂(♂“*spring1* x ♀Jester”) x ♀Jester). The Testcross progeny were grown in long day conditions and scored for flowering time. Graph showing the distribution of flowering time of plants that were classified as early flowering (n = 83) and late flowering (n = 95) compared with Jester (n = 6) and F1 plants (n = 32). The class with ≥11 nodes includes plants that had up to 21 nodes, but had not flowered by the time scoring was terminated at 69 days after germination. Plants that were “unclassified” or died young are not included. As parental and progeny plants grew at different rates, flowering was measured as the node number on the main axis at flowering.

In order to further analyse the inheritance of *spring1*, we carried out a Test cross between F1 plants from “*spring1* x Jester” and Jester [(“*spring1* x Jester*”*) x Jester]. Out of 275 progeny plants (Table 1), 83 and 95 plants were scored as early and late flowering, respectively (Fig. 3, Table 1). Thirty plants were unclassified and 67 died, confirming the lethality for a quarter of the progeny as in the previous crosses with Jester. Similar to the F2 population, a broader distribution of flowering time amongst the late class (11 - >21 nodes, Table 1) was observed in the Test cross. Nevertheless, a 1∶1 segregation ratio (χ^2^ = 0.81; 0.1<p<0.5) of early and late flowering plants was observed in the Test cross, which supports the hypothesis of a single dominant gene.

### Fine Mapping Excludes *MtCO* from the ∼0.5 Mb Interval Containing *spring1*

We reasoned that the dominance of *spring1* was unlikely to result from loss of a repressor of flowering, as this would probably confer recessive early flowering, as seen for *FLC* loss of function plants in Arabidopsis [34]. Instead, we wondered if *spring1* was a gain-of-function mutation in an activator of flowering and hypothesized that a highly active *CO* or *FT* gene conferred the *spring1* dominant early flowering phenotype. This led us to turn to a candidate gene approach for *spring1* using candidate activators as mapping markers in linkage analysis.

Previously, a major Medicago flowering time QTL in three mapping populations from different Medicago accessions was positioned on chromosome 7 and fine mapping identified a 2.4 cM confidence interval containing the QTL [15], [16], [35]. A *CO-like* gene, *MtCO,* was proposed to underly the QTL, as the gene was differentially expressed in two of the parental lines. However, the other genes in the interval (572 annotated genes) were not definitively excluded [15]. Apart from *MtCO*, five other candidate flowering time activators were located in the interval; three *FT* genes, *FTa1*, *FTa2* and *FTc* (Table S2), and two genes encoding proteins related to FD and PHYTOCHROME KINASE SUBSTRATE I (PKS) [15], [16].

The three *FT* genes are clustered together within a 33.5 kb region in the QTL interval and present on the BAC AC123593 (Table S2, Fig. S1). These all encode the key residues needed for FT function [18], [36]. Recently, we reported on a study of the Medicago *FT* genes and demonstrated that *fta1* mutants flower late and over expression of *FTa1* accelerates flowering in both Medicago and Arabidopsis, indicating that this gene is important for Medicago flowering time [18], [36]. *FTc* over expression accelerated Arabidopsis flowering, but *ftc* mutants did not have altered flowering time and over expression was not tested in Medicago. Therefore, *FTc* also is capable of promoting flowering, but may be redundant in Medicago [18]. Over expression of the third *FT* gene from the cluster, *FTa2,* did not promote Arabidopsis flowering and was not tested in Medicago, thus its role in flowering time is uncertain [18].

BLASTp analysis indicated that the *FD-like* gene on chromosome 7 is not likely to encode the orthologue of *FD*, as it is less similar to Arabidopsis FD, than are six other Medicago b-ZIP genes, but nevertheless it may still play a role in flowering time control. Similarly, the *MtCO* gene on chromosome 7 is more related to a *CO-LIKE* gene, *COL14*, which has not been shown to regulate Arabidopsis flowering, than to *CO* [15].

**Figure 4:**
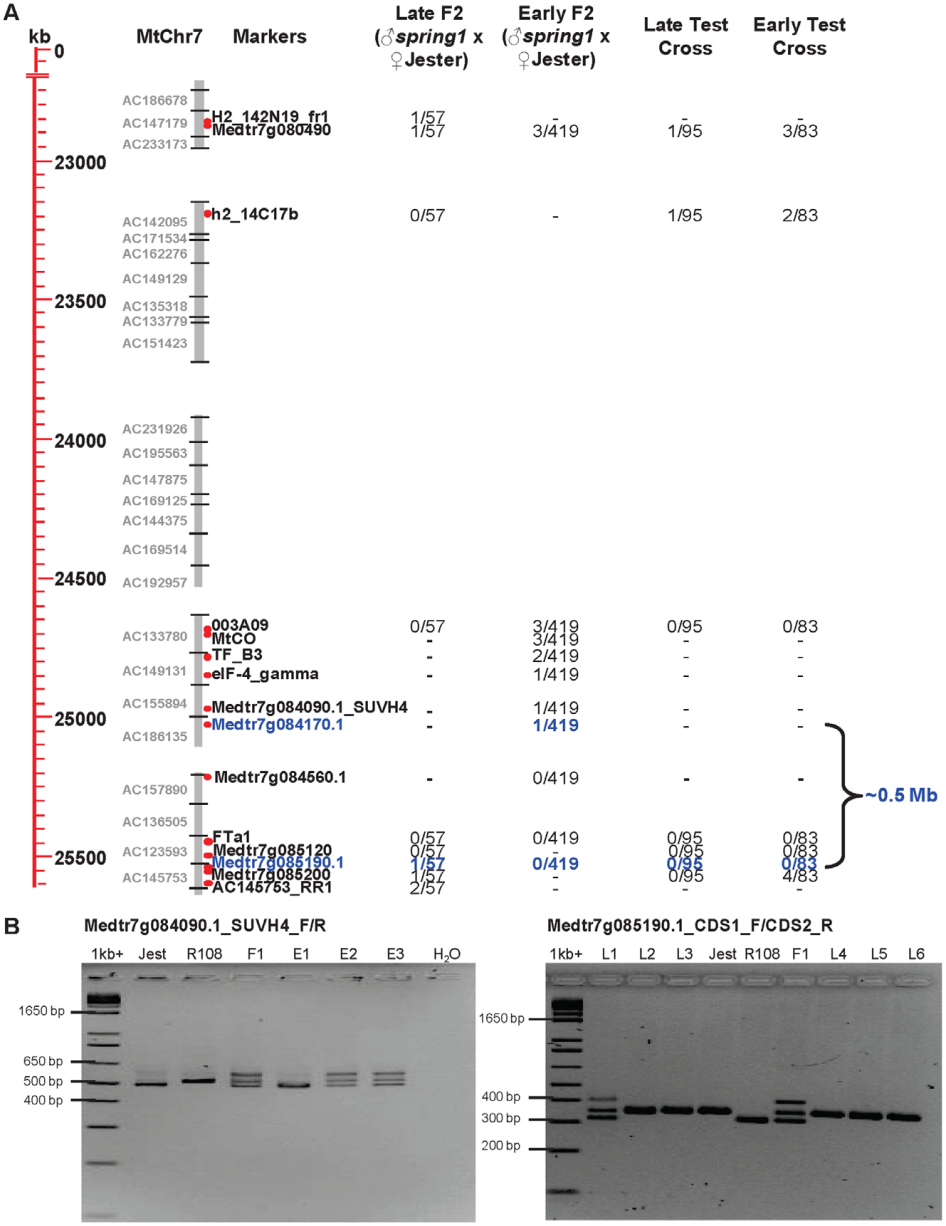
Markers for fine mapping *spring1* on chromosome 7 and defining the ∼0.5 Mb interval that contains *spring1.* a) Physical map of the *spring1* region on chromosome 7 with DNA sequence in kilobases (kb), Bacterial Artificial Chromosome (BAC) clone contigs (grey bars) and mapping marker position (red dots). Markers defining the *spring1* interval are blue. Four columns show the numbers of recombinants detected with the markers in the early and late flowering plants from the two Jester mapping populations; the F2 plants from cross “*spring1* x Jester” and the progeny of the Test cross. b) Examples of PCR genotyping using two indel DNA markers flanking the *spring1* interval. Control PCR reactions from Jester (Jest), R108 and F1 from the control cross (“R108 x Jester”) are shown. R108 and *spring1* gave the same PCR products in all cases. PCR genotyping with marker Medtr7g084090.1 (left). Products from genotyping of three early flowering F2 plants (E1 to E3) from the Mapping cross “*spring1*xJester”. Plant E1 is homozygous for the Jester band, thus Medtr7g084090.1 is separated from *spring1* by a recombination event. Genotyping with marker Medtr7g085190.1 on six late flowering F2 plants (L1 to L6) from the Mapping cross “*spring1*xJester” (right). Plant L1 is heterozygous, thus Medtr7g085190.1 is separated from *spring1* by a recombination event. A feature of both indel markers is the F1 plants and the heterozygous plants give three bands after PCR. These are the expected Jester and R108 bands and a third larger band which is likely to be a heteroduplex of the two PCR differently-sized fragments that is slightly retarded during gel electrophoresis compared to the other bands. PCR products were separated by electrophoresis on a 3% agarose gel and photographed. The Invitrogen 1 kb+ ladder provided molecular size standards. Physical maps were redrawn from a Chromosome Visualisation Tool (CViT) BLAST search http://medicagohapmap.org/with the marker sequences against the current Medicago pseudomolecule Mt3.5 genome assembly http://blast.jcvi.org/er-blast/index.cgi?project=mtbe.

In order to test if the candidate activators from chromosome 7 co-segregated with the *spring1* early flowering phenotype, we chose 29 DNA markers from the QTL interval comprised of 18 new and 11 existing markers, [15], [37] http://www.medicago.org/genome/map.php and genotyped 654 early and late flowering plants from the Mapping cross and the Test cross (Table 1). The results are summarized in Table 2 and Figure 4. The *MtCO* marker that was previously reported [15], was not able to detect DNA sequence polymorphisms between R108 and Jester, but a nearby marker 003A09 did. The marker 003A09 was very closely linked to *spring1* as no recombinants were detected with 003A09 in the 57 late flowering F2 plants or in the 178 Test cross progeny. However, our subsequent mapping with 003A09 and a newly developed *MtCO* indel marker on the 419 early flowering F2 plants, indicated that both these markers were separated by 3 recombination events from *spring1*. These 3 recombinant plants were also confirmed to be recombinant with a more distant marker Medtr7g080490. Therefore, while *MtCO* is closely linked to *spring1*, mapping excludes this gene and thus we rejected the hypothesis that a lesion in *MtCO* is causative for the *spring1* early flowering phenotype.

We also developed an indel marker for *FTa1* based on a small deletion in intron 3 that was present in both R108 and *spring1* plants, but not in Jester (Table 2). This marker showed 100% co segregation with *spring1* in the F2 and Test cross plants. *FTa1* is thus very closely linked (<0.6 cM) to *spring1*. To confirm linkage and delimit the physical interval containing *spring1*, we proceeded with linkage analysis with other markers from the *FTa1* region of chromosome 7. Two other markers from genes near *FTa1*, Medtr7g084560.1 and Medtr7g085120, also did not detect recombinants (Table 2, Fig. 4). Finally, our fine mapping located *spring1* to a physical interval on chromosome 7 of ∼0.5 Mb defined by markers Medtr7g084170.1 and Medtr7g085190.1 (Table 2, Fig. 4). Each was separated from *spring1* by a single cross over event. This region contains 78 annotated genes on two non-overlapping BAC contigs (Fig. 4, Table S2). Along with *FTa1*, the interval contains the two other *FT* genes, *FTa2* and *FTc*, but not the other candidate activators, *MtCO*, the *FD-like* and the *PKS* gene from the QTL interval [15].

### *FTa1* Transcript is more Abundant in *spring1* than R108

**Figure 5:**
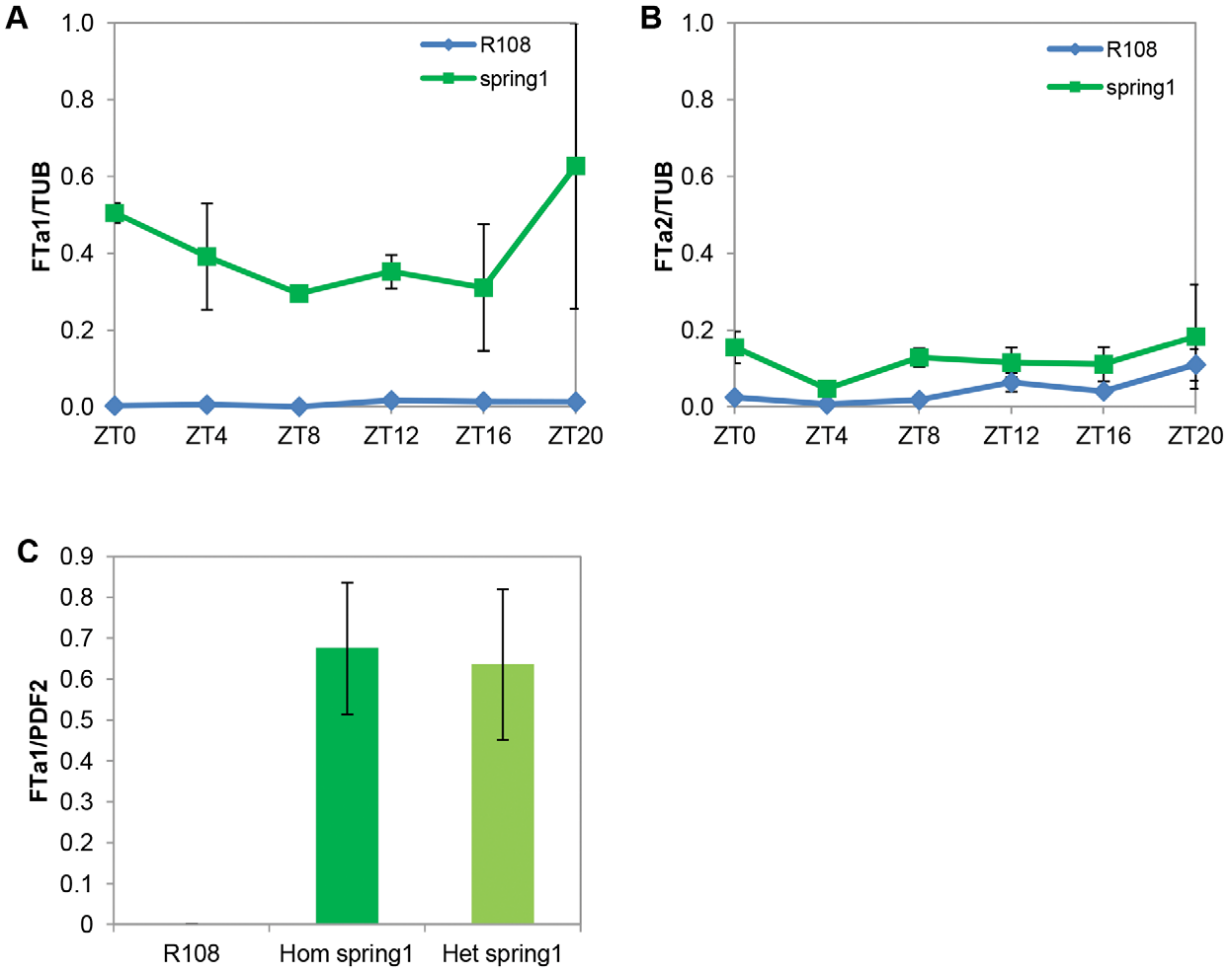
*FTa1* is up-regulated in *spring1* plants. Accumulation of *FTa1* and *FTa2* transcript in *spring1* and R108 in long day conditions was measured using qRT-PCR on 12–14 day old seedlings with two trifoliate leaves.Relative transcript abundance of *FTa1* (a) and *FTa2* (b), over a diurnal timecourse in the aerial parts of seedlings. Levels were normalised to *TUBULIN (TUB)* and calibrated relative to the expression of *FTa1* (second biological rep) at Zeitgeber 20 (ZT0 is the time of lights on). The mean +/− SE of 2 biological replicates is shown for the *spring1* samples. For R108, the two cDNA samples from each biological replicate were pooled and the mean +/− SE of the 3 technical replicates are presented. c) Accumulation of *FTa1* transcript in the first trifoliate leaf of homozygous (after two backcrosses to R108) and heterozygous *spring1* plants (F1 plants from a backcross to R108) with levels normalised to *PROTODERMAL FACTOR 2* (*PDF2)*. The mean +/− SE of 3 biological replicates is shown.

To test if any of these *FT* genes were differentially expressed in *spring1*, we harvested total aerial tissues from 12–14 day old seedlings at the two trifoliate leaf stage and analysed gene expression by qRT-PCR. The *FTa1* transcript was much more abundant in *spring1* compared to R108 at all time-points over a diurnal time course (Fig. 5a). In contrast, the *FTa2* gene was expressed at similarly very low levels in *spring1* and R108 in the diurnal time course (Fig. 5b). The expression of the third *FT* gene in the *spring1* mapping interval, *FTc*, was not detectable either in leaf or shoot apices from 12–14 day old seedlings (data not shown).

We also analysed *FTa1* expression in leaves of heterozygous *spring1* plants and saw strong up-regulation compared to R108, to the level seen in homozygous *spring1* mutants (Fig. 5c). This increased accumulation of the transcript of the flowering-time regulator *FTa1* in heterozygous plants correlated very well with the dominant early flowering phenotype of *spring1*.

### Microarray Analysis of Global Gene Expression in *spring1* and R108

To test if genes other than *FTa1* were mis-expressed in *spring1*, we compared global gene expression in *spring1* and R108 by microarray analysis. The first trifoliate leaf from 12–14 day old plants at the two trifoliate leaf stage was harvested and analysed.

**Figure 6:**
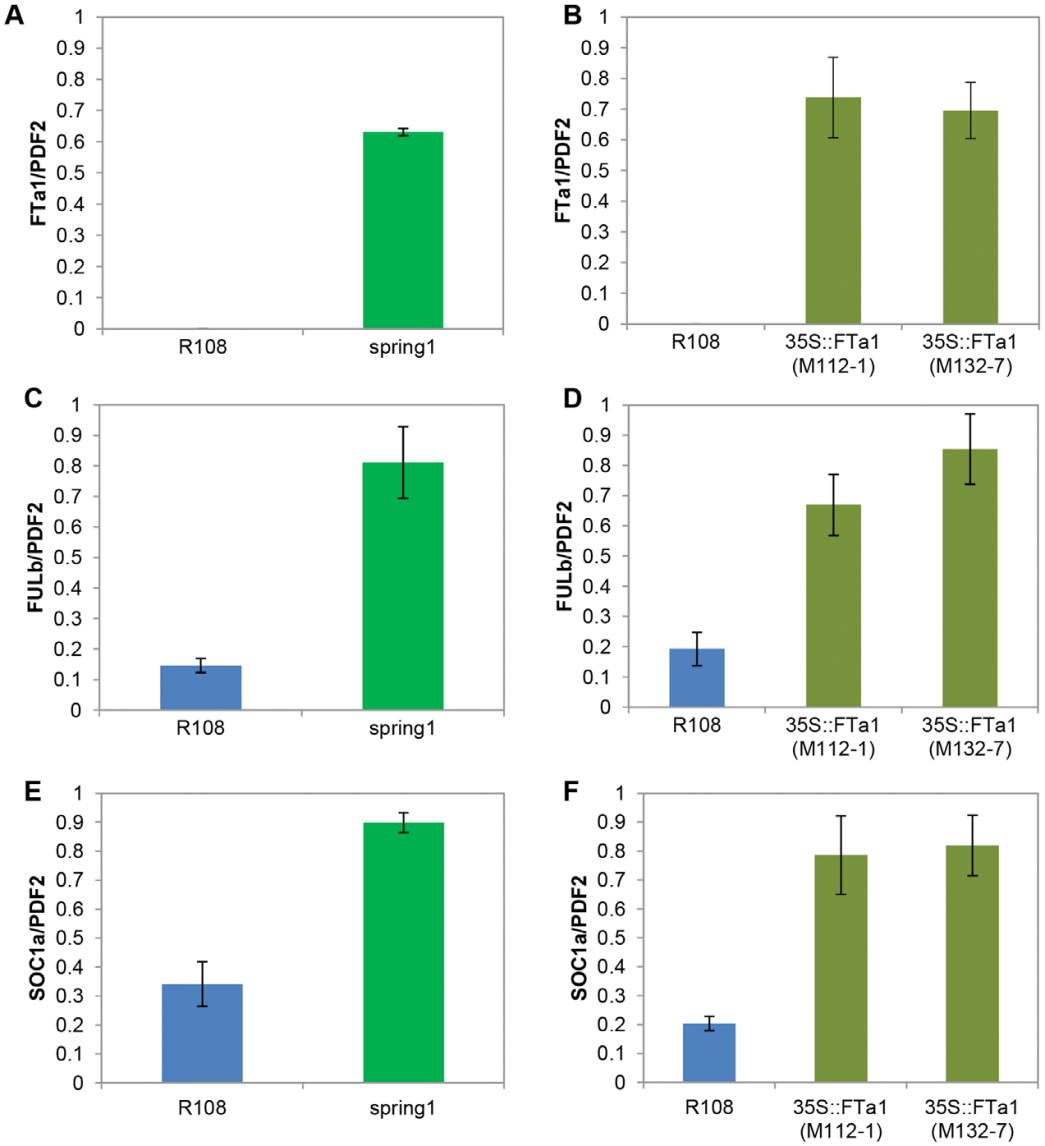
*FULb* and *SOC1a* are up-regulated in *spring1* and in transgenic Medicago plants over expressing *FTa1.* Accumulation of *FTa1, FULb and SOC1a* in *spring1, 35S::FTa1* transgenic Medicago plants and R108 in long daylength conditions was measured using qRT-PCR on the first trifoliate leaf from 12–14 day old seedlings. The mean +/− SE of 3 biological replicates is shown relative to *PDF2*. Relative transcript abundance of *FTa1* (a), *FULb* (c) and *SOC1a* (e) in *spring1.* Relative transcript abundance of *FTa1* (b), *FULb* (d) and *SOC1a* (f) in *35S::FTa1* lines.

The results indicated that the two genotypes had highly similar gene expression as only 13 genes were differentially expressed; 8 genes were up-regulated in *spring1* and 5 genes were down regulated (Table S3). Apart from *FTa1*, none of these genes was located in the *spring1* mapping interval. We carried out qRT-PCR on 12 of these genes which confirmed the microarray results (Fig. 6a, c and e, Table S3, data not shown). We also used qRT-PCR to further confirm that 7 other genes in the immediate vicinity of *FTa1,* including *FTa2* and *FTc* (Fig. S1a), were not differentially expressed in *spring1* (data not shown).

Next, we tested if the genes identified in the microarray still retained their differential expression after *spring1* had been backcrossed to R108 by carrying out qRT-PCR on homozygous *spring1* plants selected after two backcrosses (Table S3, data not shown). Eight of the 12 genes analysed, were no longer differentially expressed as before; either they were now expressed at the same level as R108 (5 genes) or had the opposite pattern of expression to that previously determined (3 genes). The ninth gene (probeset Mtr.51129.1.S1_s_at) showed very variable expression; it was undetectable by qRT-PCR in the *spring1* RNA samples that were used in the microarray, but after two backcrosses it was expressed at much higher levels than before in *spring1* (>500× higher), but still less than in R108. Three genes, *FTa1*, *FRUITFULLb* (*FULb)* and *SUPPRESSOR OF OVER EXPRESSION OF CONSTANS1a* (*SOC1a),* robustly retained their differential expression in *spring1* after the backcrosses to R108. Of these, *FTa1* was the only gene that showed 100% co-segregation with *spring1* in linkage analysis.

FULb [12] and a SOC1-like protein with 66% amino acid identity with Arabidopsis SOC1 [7], designated *SOC1a,* are *MADs* transcription factors. In *Arabidopsis, SOC1* and *FUL* function in flowering control, as a floral integrator and a floral meristem identity gene respectively, and over expression of *FT* results in an increase in the abundance of *FUL* transcripts in leaves and *SOC1* in seedlings [38]–[40]. Therefore, we reasoned that the up-regulation of *FULb* and *SOC1a* in *spring1* might result from the increased levels of *FTa1*. In order to test this, we analysed transgenic Medicago plants that were over expressing *FTa1* from the CaMV 35S promoter [18] (Figure 6b, d and f). Both *FULb* and *SOC1a* were up-regulated in the transgenic lines compared to R108. This strongly indicated that their differential expression in *spring1* resulted from the increased abundance of *FTa1*, rather than being the cause of it.

### DNA Sequence Analysis of the *FTa1* Gene in *spring1* and R108

Since *FTa1* transcript accumulation was higher in *spring1* plants and linkage mapping showed that *FTa1* co-segregated with the dominant early flowering *spring1* phenotype, we hypothesised that a change to the *FTa1* promoter in *spring1* might lead to transcriptional up-regulation of the *FTa1* gene. Therefore, to test if the *spring1 FTa1* genomic region differed in sequence from R108, we used PCR to amplify a segment of chromosomal DNA from both genotypes spanning the complete 5′ *FTa1* region from the nearest upstream gene (Medtr7g084960), to just beyond the end of the 3′UTR of *FTa1* (Fig. S1b). We obtained a 6346 bp DNA fragment from both genotypes, including the 4062 bp 5′ intergenic region, 1682 bp of the *FTa1* gene (including the three introns), the 3′UTR (349 bp) and 162 bp of the 3′ intergenic region. However, after comparing these genomic sequences we found that they were identical, showing that the difference in *FTa1* expression is not due to promoter or intron sequence changes in *spring1*.

### Conclusions

We were able to carry out a forward screen of a Medicago mutant population, identify the flowering time mutant *spring1* and fine map *spring1* to a small interval on chromosome 7. Thus we demonstrated the feasibility of mapping mutations in hybrids between *Medicago truncatula* sub species, R108 and Jemalong/A17, despite the difficulties with growth that were encountered. The *spring1* mutation confers dominant early flowering. Based on the paradigm of CO activating flowering in Arabidopsis [1], and the proposal that a *CO-like* gene, *MtCO,* might underly a major QTL for Medicago flowering [15], one idea at the outset of this work, was that a highly active CO might lead to rapid flowering in Medicago. However, fine mapping has excluded *MtCO* from the interval containing *spring1*. Nevertheless, our candidate gene mapping approach was successful as we demonstrated that another candidate activator we selected, *FTa1*, co-segregated 100% with *spring1*. We delimited a ∼0.5 Mb *spring1* interval, raising the possibility that one of the three clustered *FT* genes was responsible for the *spring1* phenotype.

Analysis of the expression of the three *FT* genes showed that one of them, the known activator of Medicago flowering, *FTa1,* was strongly up-regulated in both heterozygous and homozygous *spring1* plants. The increased abundance of *FTa1* in heterozygous *spring1* plants is consistent with the dominant early flowering conferred by the *spring1* mutation. Global analysis of gene expression in *spring1* and R108 further reinforced the linkage of *FTa1* with the *spring1* phenotype as it was the only gene mis-expressed from the mapping interval. Two of the genes that showed consistent mis-expression, encode the MADs transcription factors and candidate flowering regulators, *SOC1a* and *FULb*, both of which are also upregulated in transgenic plants over expressing *FTa1*, suggesting that their mis-expression in *spring1* results from up-regulation of *FTa1*. However, as there is no DNA sequence change in the *spring1 FTa1* promoter or introns, further work is underway to identify the genetic, or epigenetic, basis of the up-regulation of *FTa1* in *spring1*.

## Supporting Information

Figure S1 The DNA sequence of R108 and *spring1* is identical in the *FTa1* genomic region, but differs from the reference genome A17. a) Diagram showing the predicted gene annotation in the *spring1* mapping interval in the vicinity of the three *FT* genes. b) Diagram comparing the DNA sequences of the *FTa1* region between R108 and A17. PCR was used to amplify the region of DNA from the nearest upstream gene (Medtr7g084960) to just downstream of the 3′UTR of *FTa1* in *spring1* and R108. Both fragments were directly sequenced and their sequences compared to each other and to A17. The *spring1* and R108 sequences were identical. The predicted FTa1 protein encoded by A17 and R108 was identical and the three intron sequences were highly conserved, ranging from 100% nucleotide identity in the first two introns to 96% identity in the longer, third intron which has an indel of 23 bp. The 3′ UTR sequences were also highly conserved (98% identical). However, there was a striking difference in the length of the *FTa1* 5′ region, with the A17 sequence being 1347 bp shorter than the R108 sequence. This resulted from a series of indels in this region, the largest of which was a 1442 bp solo Long Terminal Repeat (LTR) from the *Angela* family of the *Ty1/copia* super family retrotransposons [41] in R108 and *spring1*, that was missing in A17. There are over 40 of this type of solo LTR in the genome [41]. There was a 5 bp repeated sequence flanking the solo LTR in R108 and *spring1*. Apart from the indels, there were blocks of high sequence conservation (94–99% nucleotide identity) shared between the 5′ region in R108 and A17.
(TIF)

Table S1 Genotyping shows segregation distortion of a DNA marker in the *spring1* interval in F2 plants of the cross of “*spring1* x Jester”. PCR genotyping using a DNA marker (*FTa1*) from the interval containing *spring1* was carried out on plant DNA samples from the two mapping crosses. These included the early and late flowering plants, but also additional samples comprising most of the “unclassified” plants and a few of the dead plants. a) In the Mapping cross “*spring1* x Jester”, our experimental hypothesis was that we expected ¼ of the plants to be homozygous for the Jester marker genotyped. However, we scored only 62 plants (1/9) as homozygous Jester out of 578 genotyped. This gives a χ^2^ value of ∼63, a value of p<0.001 leading us to reject the experimental hypothesis. b) In the Testcross, our experimental hypothesis was that we expected 1/2 of the plants to be homozygous for the Jester marker genotyped. We scored 101 plants as homozygous Jester out of 218 genotyped. This gives a χ^2^ value of ∼1.2, a value of 0.5<p<0.1 leading us to accept the experimental hypothesis.
(DOCX)

Table S2 List of annotated genes predicted within the ∼0.5 Mb interval containing *spring1.* The gene annotations were obtained from the BAC sequences in Medicago pseudomolecule Mt3.5 genome assembly http://medicagohapmap.org/. The three *FT* genes are in BAC AC123593 and shown in bold.
(DOCX)

Table S3 Microarray identification of genes that are differentially expressed in leaves of *spring1* compared to R108. The log fold change in gene expression with a p value of ≤0.05 was calculated from 3 biological repeats of each genotype grown in long day conditions. The first trifoliate leaf at the three-leaf stage was harvested. Each biological replicate was a pool of three leaves. Genes with a two-fold or greater change in gene expression are listed. ^a^All of these genes were confirmed to be differentially expressed by qRT-PCR on the RNA used in the microarray, except Mtr.21428.1.S1_at which was not done. ^b^After two backcrosses to R108, gene expression in homozygous *spring1* plants was again compared to R108, but only 3 genes, *FTa1*, *FUlb* and *SOC1a*, retained similar differential expression as first observed in *spring1*. The fourth gene Mtr.51129.1.S1_s_at showed very variable expression; it was undetectable by qRT-PCR in the original *spring1* RNA samples and after two backcrosses was expressed about 16× less than R108, but at much higher levels than before in *spring1*. After the backcrosses, the remaining genes either were expressed at the same level as R108 (^c^5 genes) or had opposite pattern of expression to that previously determined (^d^3 genes).
(DOCX)
