# Supplementary figures and images for: Fine Mapping Links the FTa1 Flowering Time Regulator to the Dominant Spring1 Locus in Medicago
Source: PLoS One. 2013 Jan 7;8(1):e53467. doi: 10.1371/journal.pone.0053467 (PMC3538541; doi:10.1371/journal.pone.0053467)

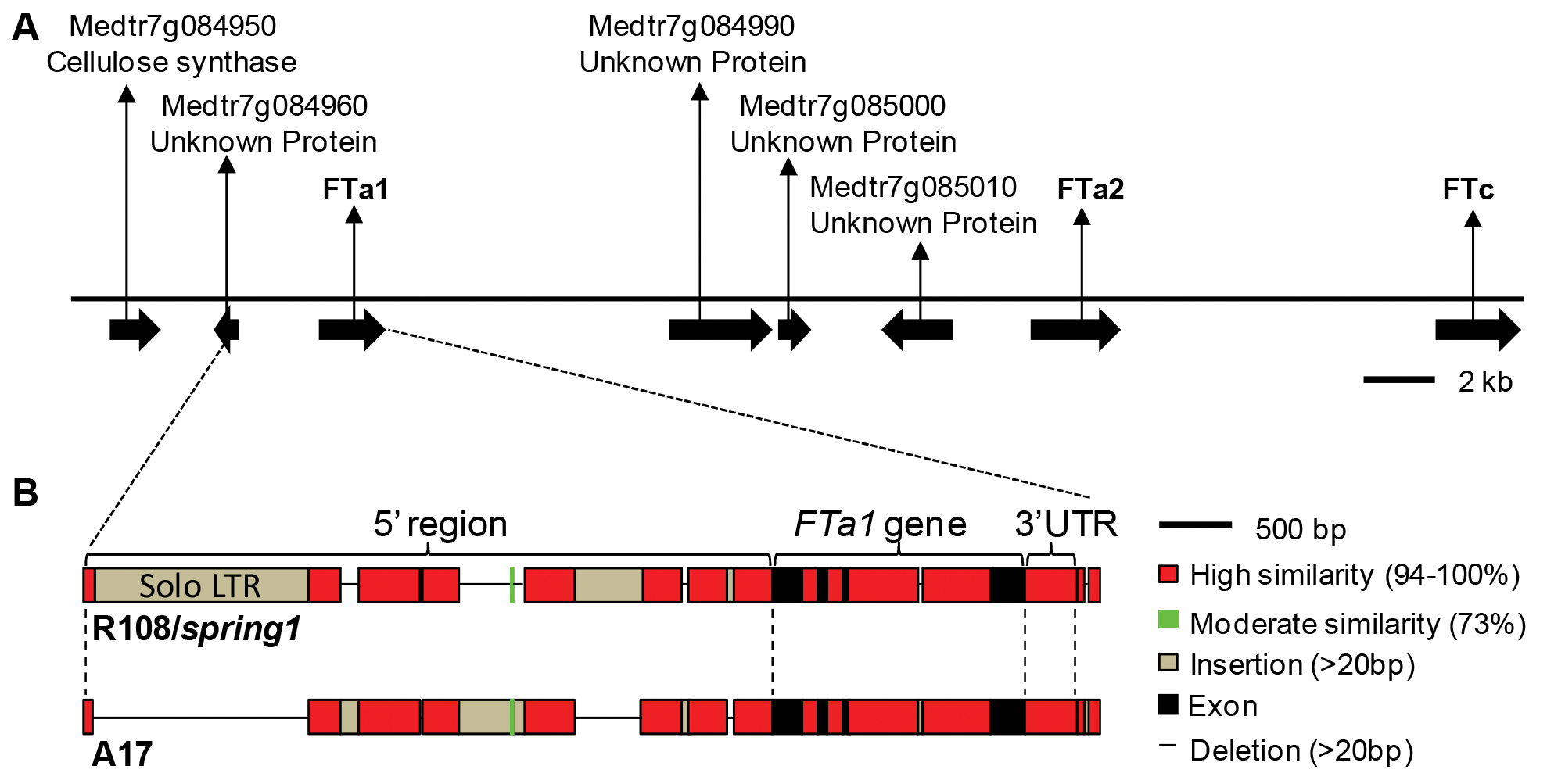

Supplement: Figure S1 — The DNA sequence of R108 and spring1 is identical in the FTa1 genomic region, but differs from the reference genome A17. a) Diagram showing the predicted gene annotation in the spring1 mapping interval in the vicinity of the three FT genes. b) Diagram comparing the DNA sequences of the FTa1 region between R108 and A17. PCR was used to amplify the region of DNA from the nearest upstream gene (Medtr7g084960) to just downstream of the 3′UTR of FTa1 in spring1 and R108. Both fragments were directly sequenced and their sequences compared to each other and to A17. The spring1 and R108 sequences were identical. The predicted FTa1 protein encoded by A17 and R108 was identical and the three intron sequences were highly conserved, ranging from 100% nucleotide identity in the first two introns to 96% identity in the longer, third intron which has an indel of 23 bp. The 3′ UTR sequences were also highly conserved (98% identical). However, there was a striking difference in the length of the FTa1 5′ region, with the A17 sequence being 1347 bp shorter than the R108 sequence. This resulted from a series of indels in this region, the largest of which was a 1442 bp solo Long Terminal Repeat (LTR) from the Angela family of the Ty1/copia super family retrotransposons [41] in R108 and spring1, that was missing in A17. There are over 40 of this type of solo LTR in the genome [41]. There was a 5 bp repeated sequence flanking the solo LTR in R108 and spring1. Apart from the indels, there were blocks of high sequence conservation (94–99% nucleotide identity) shared between the 5′ region in R108 and A17. (TIF) [file pone.0053467.s001.tif]
