# Supplementary material for: Fine Mapping Links the FTa1 Flowering Time Regulator to the Dominant Spring1 Locus in Medicago
Source: PLoS One. 2013 Jan 7;8(1):e53467. doi: 10.1371/journal.pone.0053467 (PMC3538541; doi:10.1371/journal.pone.0053467)
Supplement: Table S1 — Genotyping shows segregation distortion of a DNA marker in the spring1 interval in F2 plants of the cross of “spring1 x Jester”. PCR genotyping using a DNA marker (FTa1) from the interval containing spring1 was carried out on plant DNA samples from the two mapping crosses. These included the early and late flowering plants, but also additional samples comprising most of the “unclassified” plants and a few of the dead plants. a) In the Mapping cross “spring1 x Jester”, our experimental hypothesis was that we expected ¼ of the plants to be homozygous for the Jester marker genotyped. However, we scored only 62 plants (1/9) as homozygous Jester out of 578 genotyped. This gives a χ2 value of ∼63, a value of p<0.001 leading us to reject the experimental hypothesis. b) In the Testcross, our experimental hypothesis was that we expected 1/2 of the plants to be homozygous for the Jester marker genotyped. We scored 101 plants as homozygous Jester out of 218 genotyped. This gives a χ2 value of ∼1.2, a value of 0.5<p<0.1 leading us to accept the experimental hypothesis. (DOCX) [file pone.0053467.s002.docx]

**Supplementary Table 1 - Genotyping shows segregation distortion of a DNA marker in the *spring1* interval in F2 plants of the cross of “*spring1* x Jester”**

**A) Mapping Cross "*spring1* x Jester"**

|  | **No. Plant** | **No. Genotyped** | **Homozygous R108** | **Heterozygous** | **Homozygous Jester** | **χ² (3:1)** | **p-value** |
| --- | --- | --- | --- | --- | --- | --- | --- |
| **Early F2** | 421 | 419 | 149 | 270 | 0 |  |  |
| **Late F2** | 57 | 57 | 0 | 0 | 57 |  |  |
| **Unclassified F2** | 94 | 91 | 20 | 66 | 5 |  |  |
| **Dead F2** | 175 | 11 | 1 | 10 | 0 |  |  |
| **Total** | 747 | 578 | 170 | 346 | 62 | 62.803 | p <0.001 |

**B) Mapping "Testcross"**

|  | **No. Plant** | **No. Genotyped** | **Heterozygous** | **Homozygous Jester** | **χ² (1:1)** | **p-value** |
| --- | --- | --- | --- | --- | --- | --- |
| **Early Testcross** | 83 | 83 | 83 | 0 |  |  |
| **Late Testcross** | 95 | 95 | 0 | 95 |  |  |
| **Unclassified Testcross** | 30 | 30 | 27 | 3 |  |  |
| **Dead Testcross** | 67 | 10 | 7 | 3 |  |  |
| **Total** | 275 | 218 | 117 | 101 | 1.174 | 0.5 <p < 0.1 |
