# Supplementary material for: Fine Mapping Links the FTa1 Flowering Time Regulator to the Dominant Spring1 Locus in Medicago
Source: PLoS One. 2013 Jan 7;8(1):e53467. doi: 10.1371/journal.pone.0053467 (PMC3538541; doi:10.1371/journal.pone.0053467)
Supplement: Table S2 — List of annotated genes predicted within the ∼0.5 Mb interval containing spring1. The gene annotations were obtained from the BAC sequences in Medicago pseudomolecule Mt3.5 genome assembly http://medicagohapmap.org/. The three FT genes are in BAC AC123593 and shown in bold. (DOCX) [file pone.0053467.s003.docx]

**Supplementary Table 2 – List of annotated genes predicted within the ~0.5Mb interval containing *spring1***

| **Gene Call ID** | **Chromosome Start** | **Chromosome End** | **Function** |
| --- | --- | --- | --- |
| **BAC AC186135** | | | |
| Medtr7g084170.1 | 25012743 | 25020872 | Unknown Protein (AHRD V1) contains Interpro domain(s) IPR001750 NADH:ubiquinone/plastoquinone oxidoreductase |
| Medtr7g084190.1 | 25023946 | 25025134 | H+-transporting two-sector ATPase alpha/beta subunit central region related (AHRD V1 ***NG Q2HUB8_MEDTR) |
| Medtr7g084200.1 | 25025516 | 25032578 | LOCATED IN plasma membrane EXPRESSED IN 26 plant structures EXPRESSED DURING 15 growth stages BEST Arabidopsis thaliana protein match is aminopeptidase TAIR AT5G13940.1 Has 143 Blast hits to 138 proteins in 46 species Archae - 0 Bacteria - 59 Metazoa - 12 |
| Medtr7g084220.1 | 25040078 | 25046054 | protein kinase family protein (AHRD V1 *-*NG AT3G59110.1) contains Interpro domain(s) IPR018315 F-actin capping protein alpha subunit actin binding |
| Medtr7g084230.1 | 25047677 | 25047996 | Unknown Protein (AHRD V1) |
| Medtr7g084240.1 | 25060346 | 25061751 | polyadenylate-binding protein-related / PABP-related (AHRD V1 ***NG AT3G19330.1) |
| Medtr7g084250.1 | 25063312 | 25067079 | Nodulation receptor kinase (AHRD V1 ***NG Q8L4H4) contains Interpro domain(s) IPR005200 Glycoside hydrolase family 81 |
| Medtr7g084260.1 | 25070658 | 25071382 | H+-transporting two-sector ATPase alpha/beta subunit central region (AHRD V1 ***NG Q2HTK5_MEDTR) contains Interpro domain(s) IPR018957 Zinc finger C3HC4 RING-type |
| Medtr7g084270.1 | 25074075 | 25074585 | Unknown Protein (AHRD V1) contains Interpro domain(s) IPR001790 Ribosomal protein L10 |
| Medtr7g084280.1 | 25079436 | 25079600 | Unknown Protein (AHRD V1) contains Interpro domain(s) IPR012677 Nucleotide-binding alpha-beta plait |
| Medtr7g084290.1 | 25083319 | 25085139 | Unknown Protein (AHRD V1) contains Interpro domain(s) IPR002671 Ribosomal protein L22e |
| Medtr7g084300.1 | 25085376 | 25087544 | Chalcone synthase (AHRD V1 ***NG Q2ENC4_POPAL) |
| Medtr7g084310.1 | 25088731 | 25089748 | Unknown Protein (AHRD V1) |
| Medtr7g084330.1 | 25098078 | 25102866 | methyltransferase (AHRD V1 ***NG AT3G21950.1) contains Interpro domain(s) IPR008406 Dormancy auxin associated |
| Medtr7g084340.1 | 25104600 | 25105065 | methyltransferase (AHRD V1 ***NG AT3G21950.1) contains Interpro domain(s) IPR006186 Serine/threonine-specific protein phosphatase and bis(5-nucleosyl)-tetraphosphatase |
| Medtr7g084350.1 | 25106409 | 25108664 | S-adenosyl-L-methionine carboxyl methyltransferase family protein (AHRD V1 ***NG AT5G66430.1) contains Interpro domain(s) IPR000039 Ribosomal protein L18e |
| Medtr7g084360.1 | 25109571 | 25110486 | 1-aminocyclopropane-1-carboxylate oxidase homolog 4 (AHRD V1 *-*NG Q8H1S4) |
| **BAC AC157890** | | | |
| Medtr7g084560.1 | 25211066 | 25216571 | Helicase C-terminal Haem peroxidase plant/fungal/bacterial (Fragment) (AHRD V1 ***NG Q2HRX1_MEDTR) |
| Medtr7g084570.1 | 25216936 | 25217430 | Unknown Protein (AHRD V1) contains Interpro domain(s) IPR011989 Armadillo-like helical |
| Medtr7g084590.1 | 25228763 | 25231735 | SYP23 SYNTAXIN OF PLANTS 23 SNAP receptor (AHRD V1 ***NG AT4G17730.2) contains Interpro domain(s) IPR011710 Coatomer beta subunit C-terminal |
| Medtr7g084600.1 | 25232493 | 25232726 | Unknown Protein (AHRD V1) contains Interpro domain(s) IPR000639 Epoxide hydrolase-like |
| Medtr7g084610.1 | 25233686 | 25235025 | Unknown Protein (AHRD V1) contains Interpro domain(s) IPR000639 Epoxide hydrolase-like |
| Medtr7g084620.1 | 25237390 | 25240747 | methyltransferase (AHRD V1 ***NG AT3G21950.1) |
| Medtr7g084630.1 | 25242614 | 25243625 | Unknown Protein (AHRD V1) contains Interpro domain(s) IPR000639 Epoxide hydrolase-like |
| Medtr7g084640.1 | 25244019 | 25244664 | Unknown Protein (AHRD V1) contains Interpro domain(s) IPR000639 Epoxide hydrolase-like |
| Medtr7g084650.1 | 25246468 | 25247861 | Unknown Protein (AHRD V1) contains Interpro domain(s) IPR004330 Transcription factor FAR1-related |
| Medtr7g084660.1 | 25247902 | 25253899 | Nucleic acid-binding OB-fold (AHRD V1 *-*NG Q2HRV7_MEDTR) contains Interpro domain(s) IPR000639 Epoxide hydrolase-like |
| Medtr7g084670.1 | 25254986 | 25258704 | SNM1 SENSITIVE TO NITROGEN MUSTARD 1 (AHRD V1 ***NG AT3G26680.3) contains Interpro domain(s) IPR000639 Epoxide hydrolase-like |
| Medtr7g084680.1 | 25260239 | 25261833 | C2 domain-containing protein (AHRD V1 ***NG AT3G16510.1) contains Interpro domain(s) IPR000639 Epoxide hydrolase-like |
| Medtr7g084700.1 | 25266330 | 25266531 | Unknown Protein (AHRD V1) contains Interpro domain(s) IPR000639 Epoxide hydrolase-like |
| Medtr7g084710.1 | 25268603 | 25269754 | Unknown Protein (AHRD V1) contains Interpro domain(s) IPR008801 Rapid ALkalinization Factor |
| Medtr7g084720.1 | 25273934 | 25276630 | Unknown Protein (AHRD V1) |
| Medtr7g084730.1 | 25277008 | 25278029 | Unknown Protein (AHRD V1) contains Interpro domain(s) IPR007268 Rad9 |
| Medtr7g084740.1 | 25284467 | 25285937 | Unknown Protein (AHRD V1) contains Interpro domain(s) IPR006121 Heavy metal transport/detoxification protein |
| Medtr7g084750.2 | 25295932 | 25298584 | Xyloglucan endotransglucosylase/hydrolase (AHRD V1 ***NG Q5MB21_9FABA) |
| Medtr7g084750.1 | 25295932 | 25302578 | Xyloglucan endotransglucosylase/hydrolase (AHRD V1 ***NG O65734_CICAR) |
| Medtr7g084760.1 | 25303475 | 25305052 | Xyloglucan endotransglucosylase/hydrolase (AHRD V1 ***NG O65734_CICAR) |
| Medtr7g084760.2 | 25303475 | 25305052 | Xyloglucan endotransglucosylase/hydrolase (AHRD V1 ***NG O65734_CICAR) |
| Medtr7g084770.1 | 25306555 | 25308779 | Xyloglucan endotransglucosylase/hydrolase (AHRD V1 ***NG O65734_CICAR) |
| Medtr7g084770.2 | 25306555 | 25308779 | Xyloglucan endotransglucosylase/hydrolase (AHRD V1 ***NG O65734_CICAR) |
| Medtr7g084780.1 | 25313175 | 25313467 | Ribulose bisphosphate carboxylase large chain (Fragment) (AHRD V1 *-*NG Q95B27_PRUMU) |
| **BAC AC136505** | | | |
| Medtr7g084790.1 | 25315573 | 25317206 | Disease resistance protein RGA2 (AHRD V1 ***NG Q7XBQ9) contains Interpro domain(s) IPR002119 Histone H2A |
| Medtr7g084800.1 | 25318058 | 25321507 | Glyceraldehyde-3-phosphate dehydrogenase (Fragment) (AHRD V1 ***NG Q8VWP4_CAPAN) contains Interpro domain(s) IPR001478 PDZ/DHR/GLGF |
| Medtr7g084810.1 | 25323922 | 25328523 | WD repeat 2 (AHRD V1 ***NG Q19N38_VITVI) contains Interpro domain(s) IPR000313 PWWP |
| Medtr7g084820.1 | 25329738 | 25330662 | Nodule-specific cysteine-rich peptide 74 (AHRD V1 ***NG A7KH79_MEDTR) contains Interpro domain(s) IPR000582 Acyl-CoA-binding protein ACBP |
| Medtr7g084830.1 | 25332471 | 25333901 | Pentatricopeptide repeat-containing protein At5g59600 (AHRD V1 ***NG Q9FGR2) contains Interpro domain(s) IPR017884 SANT eukarya |
| Medtr7g084840.1 | 25338399 | 25338698 | Unknown Protein (AHRD V1) |
| Medtr7g084850.1 | 25341480 | 25350623 | Acetyl-coenzyme A synthetase (AHRD V1 ***NG A0LG91) |
| Medtr7g084860.1 | 25352459 | 25356035 | Eukaryotic release factor 1-2 (AHRD V1 ***NG D2K759_BRAOB) |
| Medtr7g084870.1 | 25356288 | 25361049 | Unknown Protein (AHRD V1) |
| Medtr7g084880.1 | 25363008 | 25382012 | importin-related (AHRD V1 *-*NG AT1G12930.1) |
| Medtr7g084890.1 | 25386891 | 25389851 | Unknown Protein (AHRD V1) |
| Medtr7g084900.1 | 25392231 | 25393068 | Unknown Protein (AHRD V1) |
| Medtr7g084910.1 | 25397454 | 25402493 | transferase family protein (AHRD V1 ***NG AT1G65450.1) |
| Medtr7g084930.1 | 25411203 | 25413909 | Unknown Protein (AHRD V1) contains Interpro domain(s) IPR000644 Cystathionine beta-synthase core |
| Medtr7g084940.1 | 25414173 | 25420059 | transferase family protein (AHRD V1 ***NG AT1G65450.1) contains Interpro domain(s) IPR001905 Ammonium transporter |
| **BAC AC123593** | | | |
| Medtr7g084950.1 | 25427126 | 25428315 | Cellulose synthase (AHRD V1 ***NG Q6YBV2_POPTM) |
| Medtr7g084960.1 | 25430047 | 25430447 | Unknown Protein (AHRD V1) contains Interpro domain(s) IPR001876 Zinc finger RanBP2-type |
| **Medtr7g084970.1** | 25432978 | 25435370 | (FTa1) FT FLOWERING LOCUS T phosphatidylethanolamine binding / protein binding (AHRD V1 **-NG AT1G65480.1) contains Interpro domain(s) IPR006172 DNA-directed DNA polymerase family B |
| Medtr7g084990.1 | 25442856 | 25445447 | Unknown Protein (AHRD V1) contains Interpro domain(s) IPR006172 DNA-directed DNA polymerase family B |
| Medtr7g085000.1 | 25445788 | 25447894 | Unknown Protein (AHRD V1) contains Interpro domain(s) IPR011989 Armadillo-like helical |
| Medtr7g085010.1 | 25448834 | 25450644 | Unknown Protein (AHRD V1) contains Interpro domain(s) IPR016038 Thiolase-like subgroup |
| **Medtr7g085020.1** | 25453016 | 25453604 | (FTa2) FT FLOWERING LOCUS T phosphatidylethanolamine binding / protein binding (AHRD V1 *--NG AT1G65480.1) contains Interpro domain(s) IPR001246 Lipoxygenase plant |
| **Medtr7g085030.1** | 25454471 | 25455236 | (FTa2) FT FLOWERING LOCUS T phosphatidylethanolamine binding / protein binding (AHRD V1 *--NG AT1G65480.1) |
| **Medtr7g085040.1** | 25463178 | 25466505 | (FTc) FT FLOWERING LOCUS T phosphatidylethanolamine binding / protein binding (AHRD V1 **-NG AT1G65480.1) contains Interpro domain(s) IPR001360 Glycoside hydrolase family 1 |
| Medtr7g085050.1 | 25467222 | 25470035 | Transmembrane and coiled-coil domain-containing protein 1 (AHRD V1 ***NG Q5R9B0) contains Interpro domain(s) IPR001360 Glycoside hydrolase family 1 |
| Medtr7g085070.1 | 25477219 | 25482200 | Aquaporin SIP1-2 (AHRD V1 ***NG Q9ATM2) contains Interpro domain(s) IPR012716 T-complex protein 1 beta subunit |
| Medtr7g085080.1 | 25482947 | 25484736 | ubiquitin family protein (AHRD V1 ***NG AT1G53930.1) contains Interpro domain(s) IPR000994 Peptidase M24 structural domain |
| Medtr7g085090.1 | 25485371 | 25492099 | ATAPY2 ARABIDOPSIS THALIANA APYRASE 2 ATPase/ nucleotide diphosphatase (AHRD V1 ***NG AT5G18280.1) contains Interpro domain(s) IPR015748 Mitogen activated protein kinase kinase kinase 3 |
| Medtr7g085100.1 | 25493457 | 25495214 | Pentatricopeptide repeat-containing protein At5g15010 mitochondrial (AHRD V1 ***NG Q9LFQ4) contains Interpro domain(s) IPR017451 F-box associated type 1 |
| Medtr7g085110.1 | 25496280 | 25497013 | Unknown Protein (AHRD V1) |
| Medtr7g085120.1 | 25499641 | 25503034 | Apyrase-like protein (AHRD V1 ***NG Q84UE3_MEDTR) contains Interpro domain(s) IPR001752 Kinesin motor region |
| Medtr7g085130.1 | 25503095 | 25503370 | Unknown Protein (AHRD V1) contains Interpro domain(s) IPR001623 Heat shock protein DnaJ N-terminal |
| Medtr7g085150.1 | 25510892 | 25514749 | ATAPY2 ARABIDOPSIS THALIANA APYRASE 2 ATPase/ nucleotide diphosphatase (AHRD V1 ***NG AT5G18280.1) |
| Medtr7g085160.1 | 25514790 | 25517500 | ATAPY2 ARABIDOPSIS THALIANA APYRASE 2 ATPase/ nucleotide diphosphatase (AHRD V1 ***NG AT5G18280.2) contains Interpro domain(s) IPR006103 Glycoside hydrolase family 2 TIM barrel |
| Medtr7g085170.1 | 25518372 | 25520241 | zinc knuckle CCHC-type family protein (AHRD V1 *--NG AT2G07760.1) |
| Medtr7g085180.1 | 25521536 | 25525824 | ATAPY1 APYRASE 1 ATPase/ calmodulin binding / nucleotide diphosphatase (AHRD V1 ***NG AT3G04080.1) |
| **BAC AC145753** | | | |
| Medtr7g085190.1 | 25526901 | 25527429 | Unknown Protein (AHRD V1) |
