# Supplementary material for: Fine Mapping Links the FTa1 Flowering Time Regulator to the Dominant Spring1 Locus in Medicago
Source: PLoS One. 2013 Jan 7;8(1):e53467. doi: 10.1371/journal.pone.0053467 (PMC3538541; doi:10.1371/journal.pone.0053467)
Supplement: Table S3 — Microarray identification of genes that are differentially expressed in leaves of spring1 compared to R108. The log fold change in gene expression with a p value of ≤0.05 was calculated from 3 biological repeats of each genotype grown in long day conditions. The first trifoliate leaf at the three-leaf stage was harvested. Each biological replicate was a pool of three leaves. Genes with a two-fold or greater change in gene expression are listed. aAll of these genes were confirmed to be differentially expressed by qRT-PCR on the RNA used in the microarray, except Mtr.21428.1.S1_at which was not done. bAfter two backcrosses to R108, gene expression in homozygous spring1 plants was again compared to R108, but only 3 genes, FTa1, FUlb and SOC1a, retained similar differential expression as first observed in spring1. The fourth gene Mtr.51129.1.S1_s_at showed very variable expression; it was undetectable by qRT-PCR in the original spring1 RNA samples and after two backcrosses was expressed about 16× less than R108, but at much higher levels than before in spring1. After the backcrosses, the remaining genes either were expressed at the same level as R108 (c5 genes) or had opposite pattern of expression to that previously determined (d3 genes). (DOCX) [file pone.0053467.s004.docx]

**Supplementary Table 3 – Microarray identification of genes that are differentially expressed in leaves of *spring1* compared to R108**

| **^a^Probesets** | **Log fold change (*spring1* vs R108)** | **Adjusted p-Value** | **Designed target/annotation** | **Forward primer**  **qRT-PCR** | **Reverse primer**  **qRT-PCR** |
| --- | --- | --- | --- | --- | --- |
| ^b^Mtr.44860.1.S1_at | 2.963 | 0.002 | MADS5 protein; FULb | AGAGCACGCAAAACTCAAGGCT | AGCTCTTTGAGACCTAAACCATCCAA |
| ^b^Mtr.14655.1.S1_at | 2.336 | 0.000 | FTa1; PEBP | GTAGCAGTAGGAATCCACTAGC | ACACTCACTCTCGGTTGATTTCC |
| ^d^Mtr.23667.1.S1_s_at | 2.304 | 0.001 | Hypothetical protein | AGAAAGAGTGCTGAAATGGATGTTG | TCGCGGATTGAGTTTCGAGCA |
| ^d^Mtr.23668.1.S1_s_at | 1.884 | 0.000 | A. thaliana genomic DNA, chr 5 | TTGGGTGAATTTATTCCTTCATCTTTC | TGAAACAAACTTTAGGCCTTGG |
| ^c^Mtr.19545.1.S1_at | 1.503 | 0.012 | hypothetical protein AC145753 | ACGCAAGTTTTCTTTCTCGCTTCCA | CCGTCGGAGGGCGATGGTTG |
| ^b^Mtr.47174.1.S1_at | 1.426 | 0.049 | MtSOC1a | GCGTTGTTCGAGCAAGAAAGAATCAGGC | GGGGCTGCTTAGAGAGCCTGGCATTT |
| ^d^Mtr.24130.1.S1_at | 1.356 | 0.034 | AC149495.15 | TCGGAGCCTCGACCTTATGCT | AGCACGCGCTCTCTCCTCCA |
| ^c^Mtr.43577.1.S1_s_at | 1.302 | 0.028 | TC95635; CYP83D1p | AGCTCTCAACGTGTCCATTCT | TCGCACCTTTTTCATCACCACCA |
| Mtr.21428.1.S1_at | -0.993 | 0.049 | hypothetical protein AC146971 | - | - |
| ^c^Mtr.31209.1.S1_s_at | -1.186 | 0.033 | Light-inducible protein ATLS1 | ACTCTACAATTGCCAACATCTTGGGTT | GCTGCTGGCTCCTCAGTTTCACC |
| ^b^Mtr.51129.1.S1_s_at | -1.964 | 0.001 | hAT dimerisation AC126009.22 | TGTGGCCTTATCTTTGGAAGAGT | TGGCTTCTTGCTCCCGAATGGT |
| ^c^Mtr.38606.1.S1_at | -3.316 | 0.000 | TC103163 Pyruvate kinase | GGATGCCGAAGTTGTTCATGAGCTG | TCCTCTGGCAACCATAGCCCCA |
| ^c^Mtr.46619.1.S1_x_at | -5.116 | 0.001 | UDP-glycosyltransferase | TCAAAGTCCGCAAAATAACTTGGCTTC | GAGTCATGACCGTGATGCTTCCAA |
